# The TatC component of the twin‐arginine protein translocase functions as an obligate oligomer

**DOI:** 10.1111/mmi.13106

**Published:** 2015-07-22

**Authors:** François Cléon, Johann Habersetzer, Felicity Alcock, Holger Kneuper, Phillip J. Stansfeld, Hajra Basit, Mark I. Wallace, Ben C. Berks, Tracy Palmer

**Affiliations:** ^1^ Division of Molecular Microbiology, College of Life SciencesUniversity of DundeeDundeeDD1 5EHUK; ^2^ Department of BiochemistryUniversity of OxfordSouth Parks RoadOxfordOX1 3QUUK; ^3^ Department of ChemistryUniversity of OxfordMansfield RoadOxfordOX1 3TAUK

## Abstract

The Tat protein export system translocates folded proteins across the bacterial cytoplasmic membrane and the plant thylakoid membrane. The Tat system in *E* *scherichia coli* is composed of TatA, TatB and TatC proteins. TatB and TatC form an oligomeric, multivalent receptor complex that binds Tat substrates, while multiple protomers of TatA assemble at substrate‐bound TatBC receptors to facilitate substrate transport. We have addressed whether oligomerisation of TatC is an absolute requirement for operation of the Tat pathway by screening for dominant negative alleles of *tatC* that inactivate Tat function in the presence of wild‐type *tatC*. Single substitutions that confer dominant negative TatC activity were localised to the periplasmic cap region. The variant TatC proteins retained the ability to interact with TatB and with a Tat substrate but were unable to support the *in vivo* assembly of TatA complexes. Blue‐native PAGE analysis showed that the variant TatC proteins produced smaller TatBC complexes than the wild‐type TatC protein. The substitutions did not alter disulphide crosslinking to neighbouring TatC molecules from positions in the periplasmic cap but abolished a substrate‐induced disulphide crosslink in transmembrane helix 5 of TatC. Our findings show that TatC functions as an obligate oligomer.

## Introduction

The targeting of proteins to their sites of cellular function is an essential process that is carried out by conserved protein sorting and translocation machineries. In prokaryotes, two major protein export pathways operate in parallel in the cytoplasmic membrane. The Sec (general secretory) machinery is a multi‐subunit complex comprising three core components that in bacteria are termed SecY, SecE and SecG (reviewed in Denks *et al*., 2014; Chatzi *et al*., 2013). SecY forms the protein transport channel (Van den Berg *et al*., 2004), to which substrate proteins can be targeted in a co‐translational manner by the signal recognition particle (Valent *et al*., 1998) or post‐translationally in bacteria by the ATPase SecA (Economou and Wickner, 1994). Substrates are targeted to the Sec machinery because they contain N‐terminal signal sequences, and they are exported in an unfolded state.

The second major protein transport system found in prokaryotes is the Tat (twin‐arginine) translocase, which transports fully folded proteins across the cytoplasmic membrane (reviewed in Palmer and Berks, 2012; Frobel *et al*., 2012; Berks, 2015). Substrates of the Tat pathway also contain N‐terminal signal sequences, which, although related to Sec signal peptides, differ in overall hydrophobicity and because they contain an almost invariant twin‐arginine motif (Berks, 1996; Cristobal *et al*., 1999; Stanley *et al*., 2000). The Tat machinery is made up of membrane proteins of the TatA and TatC families (Settles *et al*., 1997; Bogsch *et al*., 1998; Sargent *et al*., 1998; Yen *et al*., 2002; Jongbloed *et al*., 2004). In *Escherichia coli*, and most Gram negative bacteria, two TatA family proteins, TatA and TatB, assume distinct roles in the Tat protein export pathway (Sargent *et al*., 1998; 1999). The TatB protein forms a stable complex with TatC, together acting as the receptor for Tat substrates (e.g. Bolhuis *et al*., 2001; Cline and Mori, 2001; Richter and Bruser, 2005; Alami *et al*., 2003; Tarry *et al*., 2009). By contrast, the TatA protein is found as small homomultimeric units that assemble into larger oligomers driven by the protonmotive force and interaction with the substrate‐bound TatBC complex (Mori and Cline, 2002; Alami *et al*., 2003; Dabney‐Smith *et al*., 2006; Leake *et al*., 2008; Alcock *et al*., 2013; Rose *et al*., 2013). Both Sec and Tat machineries are also found in the thylakoid membranes of plant chloroplasts (Celedon and Cline, 2013).

The oligomeric state that the SecYEG channel adopts during protein translocation has long been controversial. Numerous lines of evidence have shown that purified SecYEG complexes in detergent solution, or following reconstitution into liposomes, are dimeric or even tetrameric (e.g. Breyton *et al*., 2002; Bessonneau *et al*., 2002; Manting *et al*., 2000; Mitra *et al*., 2005). Although a protein conducting channel is present in the SecYEG monomer (Van den Berg *et al*., 2004), it has been proposed that dimerisation of SecYEG is required, for example, to activate SecA (Osborne and Rapoport, 2007; Deville *et al*., 2011; Dalal *et al*., 2012). However, others have shown that a reconstituted SecYEG monomer is functional for protein translocation (Kedrov *et al*., 2011) and an *E. coli* SecY variant that cannot support dimerisation can replace wild‐type SecY *in vivo* (Park and Rapoport, 2012). None‐the‐less, dimers of SecYEG have been detected at endogenous levels in *E. coli* (Boy and Koch, 2009). Recently, a heteromultimeric complex of monomeric SecYEG with SecDF–YajC–YidC, termed the holo‐translocon, has also been purified that is particularly proficient for membrane protein insertion (Schulze *et al*., 2014). It has been proposed that SecYEG undergoes dynamic exchange between homodimers and holo‐translocons as a means to modulate translocation activity.

The oligomeric state of the Tat protein translocase is also uncertain. A wide range of oligomeric states for the TatA protein, both *in vitro* and *in vivo*, have been reported (Gohlke *et al*., 2005; Oates *et al*., 2005; Dabney‐Smith *et al*., 2006; Leake *et al*., 2008), with multimerisation of TatA into large assemblies being associated with protein translocation (Dabney‐Smith *et al*., 2006; Dabney‐Smith and Cline, 2009; Alcock *et al*., 2013; Rose *et al*., 2013). The TatBC receptor complex is also multimeric in detergent solution (e.g. Cline and Mori, 2001; Bolhuis *et al*., 2001), even at native levels of expression (McDevitt *et al*., 2006), and multimers of TatB and TatC have also been detected *in vivo* (Alcock *et al*., 2013; Rose *et al*., 2013). Analysis of purified TatBC complexes by negative stain electron microscopy shows that they contain more than one species, probably with different numbers of TatB and TatC subunits (Oates *et al*., 2003; Tarry *et al*., 2009), and some level of heterogeneity has also been seen for the *E. coli* TatBC complex analysed by blue‐native polyacrylamide gel electrophoresis (BN‐PAGE) (e.g. Behrendt *et al*., 2007; Orriss *et al*., 2007).

Under some circumstances, the TatBC complex also exhibits functional multivalency. For example, single TatBC complexes from *E. coli* can be purified with up to two bound substrate proteins (Tarry *et al*., 2009), and in *Salmonella enterica* a heterodimer of the tetrathionate reductase A and B subunits, each with their own Tat signal sequence, are co‐translocated as a complex where each signal sequence is likely to engage different TatBC subunits (James *et al*., 2013). In plant thylakoids up to four thylakoid substrate precursor proteins tethered to each other by disulphide bonds can be exported in a single step (Ma and Cline, 2010). However, it is not clear whether the TatBC complex is an obligate multimer, or, like SecYEG, can retain activity if oligomeric interactions are disrupted.

Here we have addressed the question of whether the TatBC complex is a functional oligomer by undertaking a genetic screen to isolate mutations of *tatC* that prevent function of the Tat system even when a wild‐type copy of *tatC* is present. The isolation of such mutations confirms that oligomerisation of TatC is essential for operation of the Tat pathway. Biochemical analysis of TatBC complexes harbouring individual amino‐acid substitutions that confer dominant negative activity shows that the complexes are of lower oligomeric state than the native TatBC complex and that the substitutions block substrate‐induced recruitment of TatA.

## Results

### Isolation of dominant negative mutants of *E* *. coli* tatC

Crosslinking studies have shown that within the TatBC complex, neighbouring TatC proteins are in close proximity (e.g. Punginelli *et al*., 2007; Zoufaly *et al*., 2012; Aldridge *et al*., 2014). Moreover, a covalent TatC dimer is stable and supports full Tat activity (Maldonado *et al*., 2011a), and when produced in the absence of other Tat components, TatC is able to self‐interact to form multimers (Behrendt *et al*., 2007; Orriss *et al*., 2007). However, the structure of the isolated TatC protein from *Aquifex aeolicus* in three different crystallisation environments does not identify physiologically plausible dimer contacts (Rollauer *et al*., 2012; Ramasamy *et al*., 2013). Therefore, to assess whether oligomerisation of TatC is functionally required for Tat activity, and if so to identify potential sites of self‐interaction, we undertook a screen for dominant negative mutations of *tatC*.

The premise of the screen is that, if TatC is a functional multimer, it should be possible to identify variants of TatC that can still interact with the wild‐type protein such that they prevent the wild‐type copy from working. To this end, we screened a pre‐existing random library of *tatC* mutations present in plasmid pTAT1d (which has a ColE1 origin and a copy number of around 20 per cell), which also harbours the *tatA* and *tatB* genes (Maldonado *et al*., 2011b; Kneuper *et al*., 2012). The mutant library comprises approximately 600,000 individual clones with an average of 2.5 errors per *tatC* gene (Kneuper *et al*., 2012). Screening was undertaken in strain MC4100, which encodes the native chromosomal copy of *tatABC*. The first round of screening utilised a positive selection for loss of Tat function based on the export of chloramphenicol acetyltransferase (CAT) fused to the *E. coli* TorA Tat signal peptide (Maldonado *et al*., 2011b). Since CAT is only active in the cytoplasm because it requires a source of acetyl CoA, export by the Tat system renders cells sensitive to growth in the presence of chloramphenicol, whereas inactivation of Tat function provides resistance. We screened more than 2 million transformants in MC4100 and found approximately 4000 that could grow in the presence of 200 μg ml^−1^ chloramphenicol.

Next we took a subset of 700 of the chloramphenicol‐resistant transformants and subjected them to two additional phenotypic screening tests that also assess Tat functionality. Screening in the presence of 2% SDS assesses the ability of *E. coli* to export the Tat‐dependent amidase enzymes, AmiA and AmiC, that are involved in cell wall remodelling (Buchanan *et al*., 2002; Ize *et al*., 2003), while screening for anaerobic growth with trimethylamine‐*N*‐oxide (TMAO) as sole electron acceptor assesses whether the Tat‐dependent TMAO and dimethylsulphoxide reductases have been exported (Sargent *et al*., 1998; Weiner *et al*., 1998). From this we found 97 clones that were defective for growth on one or both of these selective media (Tables 1 and 2).

**Table 1:** Amino‐acid sequence changes encoded in dominant negative clones isolated from the *tatC* mutant library

Clone	Growth on TMAO	Growth on SDS	TatC substitution/s
9	−	−	T6I P131L^a^ N139T D150N^b^ W180L T184I
50	−	+/−	**D5E** A160V
62	−	+/−	V203D G204E^b^
72	+/−	+/−	F37S
77	−	−	S114R S153C **F165L** G199R G238S
85	−	+/−	**D5N** M59I^b^ D63V^a^ Y84C
95	+/−	+/−	**S66P** a K255stop
128	+/−	+/−	R17P P67L
133	−	+/−	**S66L** b **K73E** E103V^b^ F232S
147	+/−	+/−	L137H^a^ I151V^b^ V202I
151	−	+/−	**S66P** a A96V
162	−	−	**M59K** a **K73E** A133V
175	+/−	+/−	**P54S D150G** a
182	−	+/−	I60V^b^ **S66L** b
191	+/−	+/−	**D150Y** b
242	+/−	−	V29A A61D I151T^a^ F235S
260	−	+/−	**S66P** a K192N
266	−	+/−	L21P S57P
287	−	+/−	Q52L **D150G** a
291	−	+/−	I41V **S66P** a L197Q
298	−	+/−	D4N **S66P** a
318	−	−−	L53F **V64E** a K101R M122R M181T E244V
320	−	−−	V78E H102L S148P^a^
328	−	−	**V64E** a F69S L161P
335	−	−	**S66L** b F134Y
365	−	+/−	S2T I28T T70K^b^ L74P^a^ L99Q^a^ E143V C224S
386	−	+/−	L21P **P48L** a
394	−	+/−	L21P K51R **S66P** a
404	−	+/−	**D5Y T11S S66L** b V147A^b^ E254G
422	−	−	**V64E** a F124I
429	−	+/−	M77K P85L S113P A123V S233L
432	+/−	+/−	S148P^a^
444	−	+/−	**V64E** a
457	−	+/−	C23Y^b^ L111Q P142L F157I
464	+/−	+/−	**T11A** L137P^b^ A141S **D150Y** b Y154H R234C
465	−	+/−	Δ151I^b^ Δ152A
469	−	+/−	F130I **D150Y** b F169S I220T Y223C
471	−	+/−	**S66P** a G204W^b^
477	−	+/−	**P48Q** b **S66P** a V173I
478	−	+/−	**M59K** a **V64E** a K101R V237A
483	+/−	+/−	L88P M205I^b^
497	+/−	+/−	**S66P** a A152V
505	+/−	+/−	**S66P** a
508	+/−	+	**P48S** a **S66P** a M122V
514	−	+	I60F **S66P** a F165I
517	+/−	+	**S66P** b L111R V237A
520	−	+	S57L **S66P** a
521	+/−	+	F31Y **P54L** G144R
554	−	+	R17H A47P G135S L161Q D188G F235L R241Q
578	−	−	Q52L **D150Y** b
586	+/−	+/−	V147E^a^ **D150G** a F226S E254G
594	+/−	+/−	**P48L** a **M59V** b S79P V158A G199S
608	+/−	+	V86M F134S
611	+/−	−	L137H^a^ S148P^a^
612	+/−	+	**S66P** a S83P
621	+/−	+	L34P A61V F76L Q90P
626	−	−	V145G^b^ A164T
644	+/−	−	**V64E** b R105H M181K
651	−	−	I60T^b^ V145E^a^ V171M
664	−	−	**P48L** a F157S **F165L** L197Q
668	+/−	+	I183F G229D
674	+/−	−	**S66P** a **K73R** A84V H102L G135S
679	+/−	−	**S66P** a R190C
680	−	−	**P54S** S57L F69S L74P^a^ G144R
689	−	−	**P54L** F69L T70K E245D
690	+/−	−	N22I **S66P** a
693	+/−	−	L34Q **S66P** a A200V R243Q
699	−	−	A61D; K73E; F134S
702	+/−	−	**S66P** a V230D
706	+/−	−	**T11K D150Y** b A152V **F165I**
708	+/−	−	H12L A47V **V64M** b **S66L** b H102L
711	+/−	+/−	G182R L207P L225P^a^
723	−	−	L13P **S66P** a V147A^b^
726	+/−	−	**D5G T11A** I228 frameshift
729	−	−	**T11M S66P** a T70M^b^ G135S A164T R190H V202I
735	−	−	**P48L** a D63V^a^
743	−	−	L20P^a^ F136L **D150G** a

‘+’ indicates growth, ‘ + /−’ poor growth and ‘–’ no growth on the indicated selective medium. Substitutions in boldface are where the indicated residue was found substituted on four or more occasions in the isolated clones.

a Identical substitutions that have been previously isolated that are known to inactivate the function of TatC (Kneuper *et al*., 2012).

b Substitutions at these amino‐acid positions are known to inactivate TatC when substituted to something other than that identified here.

**Table 2:** Amino‐acid sequence changes encoded in dominant negative clones found in the context of fused TatBC isolated from the *tatC* mutant library

Clone	Growth on TMAO	Growth on SDS	TatC substitution/s in addition to fused TatB–TatC
1^a^	−	+/−	L49stop
5^b^	−	+/−	E187stop
6^a^	−	−	P8T I24F P54S D150N A152V K191 stop
74^c^	−	−	P67L W180 stop
75^d^	−	+/−	P186L Y195 stop
91^e^	−	+/−	I50V M205V
189^f^	−	+/−	V198 frameshift
193^b^	−	+/−	L20Q F69L L218 stop
322^b^	−	+/−	A123G T140A Q215 stop
340^g^	−	+/−	V230I A249V
343^d^	−	−	F37S S113T A141V S214P E227V
349^h^	−	+/−	H12R D150G S153N
355^i^	−	+/−	Q55L F127Y V202I L218 stop
358^f^	−	−	A160V L189 stop
364^i^	−	−	F169I S185 stop
366^d^	−	+/−	I30T V171A T208M E246G
370^i^	−	−	N139I Y195 stop
397^f^	−	−	C224R
409^b^	−	+/−	S83T P109S L218 stop
410^b^	−	+/−	Y42 stop

The amino‐acid sequences at the TatB‐C fusion junction are as follows (each one starting at S166 of TatB and ending at S2 of TatC):

a SSSINLSMS.

b SSSDNLSMS.

c SSSDTLSMS.

d SSSVTLSMS.

e SSSVNLSMS.

f SSSDKLSMS.

g SSSITLSMS.

h SSSKTLSMS.

i SSSETLSMS.

The sequence of the plasmid‐encoded *tatABC* genes in each of these strains was subsequently determined, and the *tatC* alleles fell into two broad categories. The first, shown in Table 1, had one or more point mutations within the *tatC* sequence but, with the exception of clone #726, encoded a full‐length TatC. The second (Table 2) had mutations leading to the generation of a TatBC fusion protein along with additional mutations, frequently premature stop codons, within the *tatC* gene.

### Individual dominant negative substitutions in TatC cluster in the periplasmic cap

We noted from Table 1 that although substitutions were found throughout TatC, there was a strikingly high number in the first and second periplasmic loops. For example, there were 4 substitutions each at P54, M59, K73, 6 at P48 and 27 at S66 in the first periplasmic loop, and 10 at D150 in the second periplasmic loop. These loop regions were previously found as hotspots for substitutions that inactivate the function of TatC (Kneuper *et al*., 2012) and form a stabilising cap over the transmembrane helices of the protein (Rollauer *et al*., 2012; Ramasamy *et al*., 2013). By contrast, substitutions in other regions of TatC that have previously been highlighted by mutagenesis to have essential roles in TatC function, for example, the first cytoplasmic loop that is involved in signal peptide binding (Strauch and Georgiou, 2007; Rollauer *et al*., 2012), were not over‐represented among the mutants isolated.

To determine which of the substitutions listed in Table 1 led to the dominant negative activity of TatC, we re‐introduced substitutions individually in TatC encoded on plasmid pTAT1d along with *tatA* and *tatB*. In doing so, we selected all residues in which substitutions occurred four or more times in Table 1, and in addition, we remade all of those clones that contained only a single substitution. Finally, as additional controls, we also included previously identified substitutions that are known to inactivate the function of TatC that fall (a) within the periplasmic loops (L74P – also found twice in Table 1, and V145E – also found once in Table 1; Kneuper *et al*., 2012) and (b) elsewhere on the TatC protein (F94R, G204R, M205R, D211R and D215R; Kneuper *et al*., 2012). All of the single substitutions tested are listed in Table 3.

**Table 3:** Single amino‐acid substitutions in TatC that were tested for activity and dominance

D5E	P54L	K73E	S148P	G204R	C224R^a^
T11A	M59K	L74P	D150G	M205R	
F37S	V64E	F94R	D150Y	D211R	
P48L	S66P	V145E	F165L	Q215R	

a This substitution was found in conjunction with fused TatBC (Table 2).

**Figure 1:**
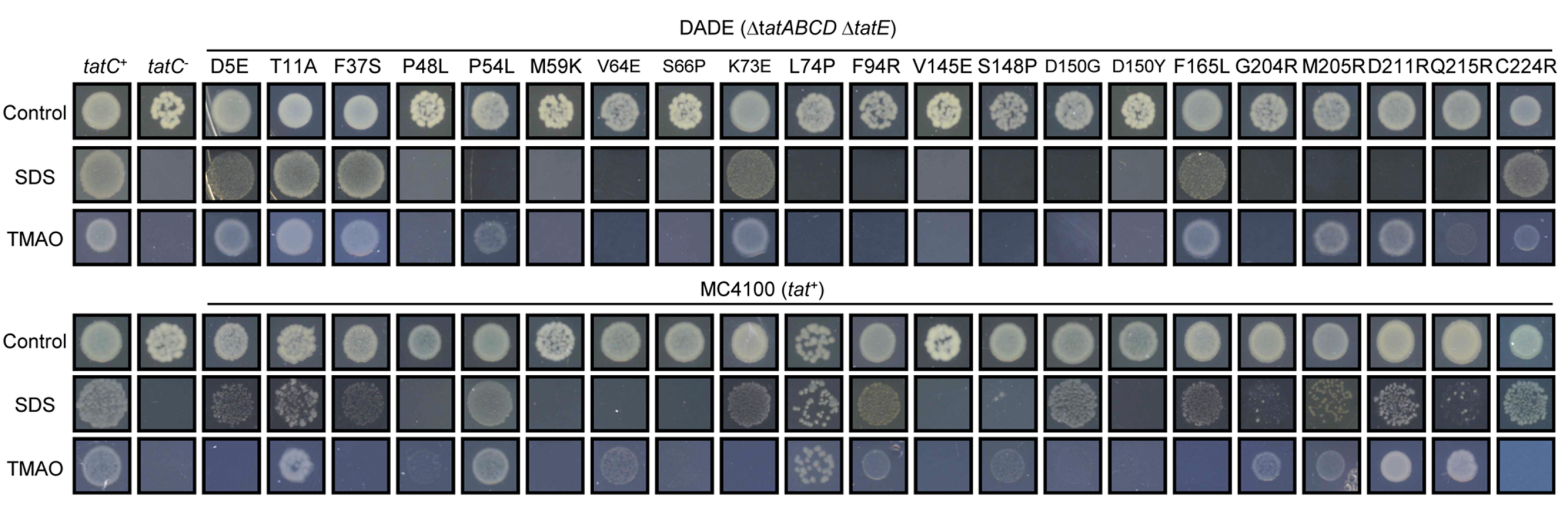
Phenotypic characterisation of TatC single amino‐acid variants in a *tatC* ^−^ and *tatC* ^+^ strain background. Spot tests of strain DADE (Δ*tat* *ABCD*, Δ*tatE*; top panel) or MC4100 (*tat* ^+^; bottom panel) harbouring the pTAT1d vector encoding TatA, TatB and the indicated amino‐acid variant in TatC. Strain MC4100 also harboured the additional screening plasmid pTTC1 (since we noted that dominant negative phenotypes were more clear‐cut in the presence of the overproduced TorA–CAT fusion protein). *tatC* ^+^ indicates strain DADE harbouring wild‐type pTAT1d (top panel) or strain MC4100 harbouring pTTC1 and wild‐type pTAT1d (bottom panel) and *tatC* ^−^ is strain DADE harbouring the pTAT1d parental plasmid, pUniprom. Strains were grown overnight in liquid media, diluted to give an OD_600nm_ of 0.0001 and 5 μl aliquots were replica spotted onto: LB medium (control), LB medium containing 2% SDS or M9 medium containing glycerol and TMAO. Strains were incubated for 3 days aerobically at room temperature for growth on LB and LB–SDS containing medium and anaerobically at 37°C for growth on M9–glycerol–TMAO medium.

**Figure 2:**
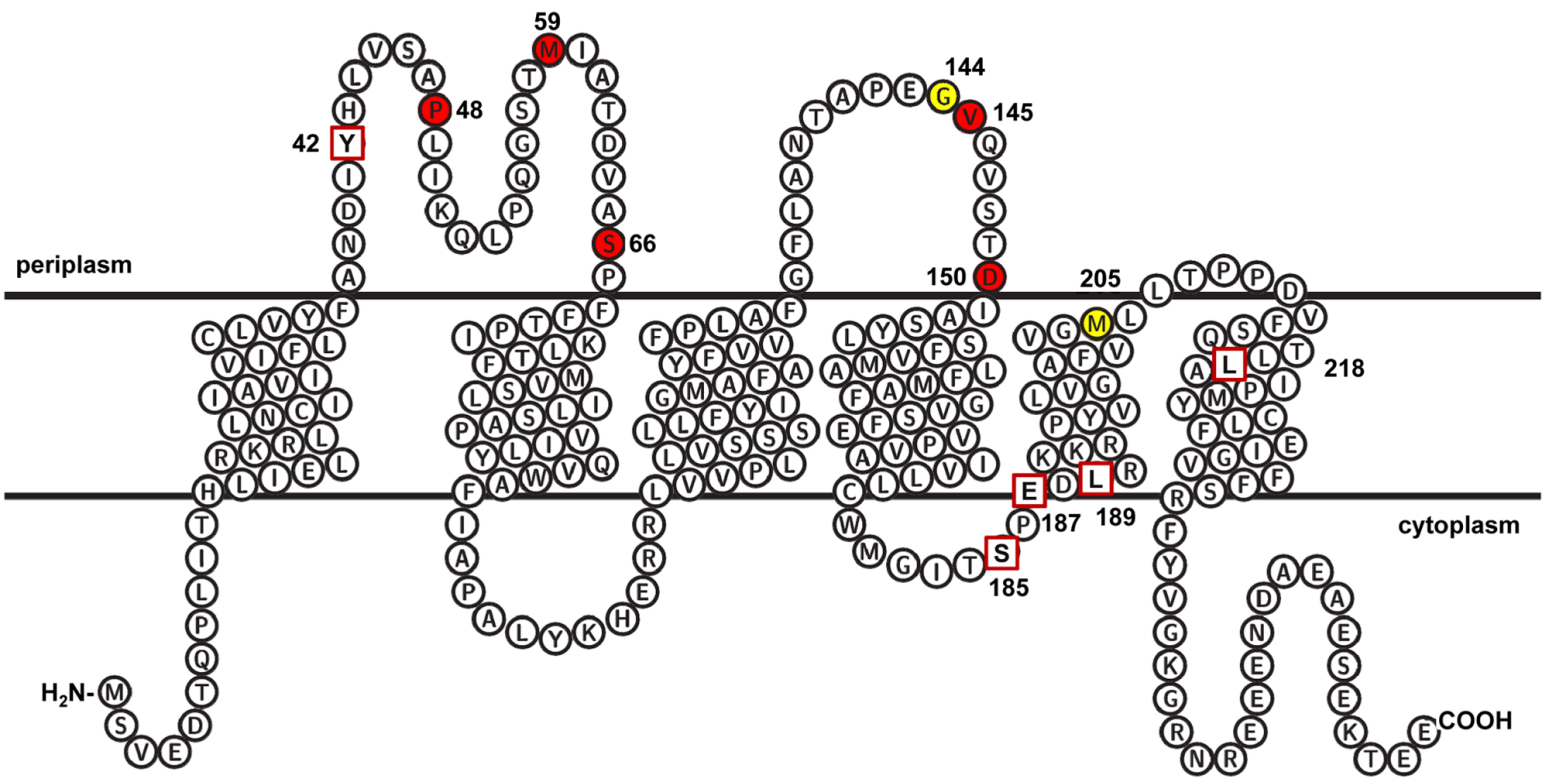
Topological organisation of TatC and location of TatC variants analysed in this study. The diagram was generated using TEXtopo (Beitz, 2000) and the positions of the transmembrane helices are indicated and single amino‐acid residues that when substituted give rise to a dominant negative phenotype are shown in red. The red boxes indicate the positions of truncations that, in the context of a TatB–TatC fusion, give rise to dominant negative activity. Residues G144 and M205 that were mutated to cysteine and used for disulphide crosslinking analysis are shown in yellow.

Each plasmid‐encoded substitution was introduced into strain background DADE (which lacks all chromosomal *tat* genes, allowing us to score whether the substitution inactivates the function of TatC) and into MC4100 (which is *tat* ^+^, thus allowing us to score for dominance). Tat function was then assessed using selective media containing SDS or TMAO. As shown in Fig. 1 (top panel), most of the individual substitutions tested severely affected the function of TatC when each variant was produced as the sole TatC copy by preventing growth on at least one of the two selection media, with the exception of the D5E, T11A, F37S, K73E, F165L and C224R substitutions. The TatC positions and nature of the TatC inactivating substitutions were almost all previously identified by Kneuper *et al*. (2012), although two novel TatC inactivating substitutions, P54L and D150Y, were found. However, very few of these TatC substitutions led to an inactive Tat system in the presence of wild‐type *tatC* (Fig. 1, bottom panel). Indeed only five single TatC substitutions falling in either the first (P48L, M59K and S66P) or second (V145E and D150Y) periplasmic loop prevented growth of the wild‐type strain MC4100 on both SDS‐ and TMAO‐containing media. We conclude that single dominant negative amino‐acid substitutions are strongly and almost exclusively associated with the periplasmic cap of TatC. The positions of these dominant negative TatC amino‐acid substitutions are shown in Fig. 2 on a schematic representation of TatC.

### TatBC fusions show dominant negative activity when combined with additional stop codon substitutions

A second class of TatC variant isolated after screening for dominant negative mutations was those that encoded a TatBC fusion protein along with other substitutions, particularly stop codons. To determine which mutations were responsible for the dominant negative behaviour of these clones, we dissected out some of the individual mutations found in clones 5, 75, 358, 364, 397 and 410 listed in Table 2, and we also assessed the effect of the Y42 stop, S185 stop, E187 stop, L189 stop and L218 stop mutations that co‐occurred along with a TatBC fusions encoded by clones in Table 2. The mutations that we tested are listed in Table 4 and the positions of the stop codon substitutions are also shown on a schematic of TatC in Fig. 2.

**Table 4:** TatBC fusions and TatC truncations tested for dominance in this study

Clone name	TatBC fusion sequence/TatC substitution
TatBC fusion2	*^tatB^* ^166^SSSDNLSMS*^tatC^* ^2^
TatBC fusion6	*^tatB^* ^166^SSSDKLSMS*^tatC^* ^2^
TatBC fusion9	*^tatB^* ^166^SSSETLSMS*^tatC^* ^2^
TatCY42 stop	No fusion, TatC Y42 stop
TatCS185 stop	No fusion, TatC S185 stop
TatCL189 stop	No fusion, TatC L189 stop
TatCE197 stop	No fusion, TatC E197 stop
TatCL218 stop	No fusion, TatC L218 stop
Fusion2Y42	*^tatB^* ^166^SSSDNLSMS*^tatC^* ^2^, TatC Y42 stop
Fusion2E187	*^tatB^* ^166^SSSDNLSMS*^tatC^* ^2^, TatC E187 stop
Fusion2L218	*^tatB^* ^166^SSSDNLSMS*^tatC^* ^2^, TatC L218 stop
Fusion6A160V	*^tatB^* ^166^SSSDKLSMS*^tatC^* ^2^, TatC A160V
Fusion6L189	*^tatB^* ^166^SSSDKLSMS*^tatC^* ^2^, TatC L189 stop
Fusion6A160VL189	*^tatB^* ^166^SSSDKLSMS*^tatC^* ^2^, TatC A160V, L189 stop
Fusion6AC224R	*^tatB^* ^166^SSSDKLSMS*^tatC^* ^2^, TatC C224R
Fusion9F169I	*^tatB^* ^166^SSSETLSMS*^tatC^* ^2^, TatC F169I
Fusion9S185*	*^tatB^* ^166^SSSETLSMS*^tatC^* ^2^, TatC S185 stop
Fusion9 F169I S185*	*^tatB^* ^166^SSSETLSMS*^tatC^* ^2^, TatC F169I, S185 stop

**Figure 3:**
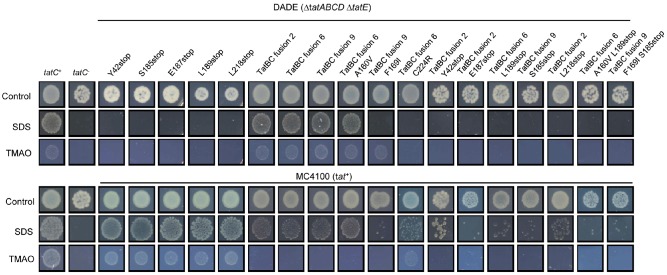
Phenotypic characterisation of TatBC fusions and TatC truncations in a *tatC* ^−^ and *tatC* ^+^ strain background. Spot tests of strain DADE (Δ*tat* *ABCD*, Δ*tatE*; top panel) or MC4100 (*tat* ^+^; bottom panel) harbouring the pTAT1d vector that contains the mutated *tat* *ABC* operon encoding TatC variants or TatB‐C fusion proteins, as indicated. Strain MC4100 also harboured the additional screening plasmid pTTC1. *tatC* ^+^ indicates strain DADE harbouring wild‐type pTAT1d (top panel) or strain MC4100 harbouring pTTC1 wild‐type pTAT1d (bottom panel) and *tatC* ^−^ is strain DADE harbouring the empty vector pUniprom. Strains were grown overnight in liquid media, diluted to give an OD_600nm_ of 0.0001 and 5 μl aliquots were replica spotted onto: LB medium (control), LB medium containing 2% SDS or M9 medium containing glycerol and TMAO. Strains were incubated for 3 days aerobically at room temperature for growth on LB and LB–SDS containing medium and anaerobically at 37°C for growth on M9–glycerol–TMAO medium.

We found that, as expected, the TatC stop codon substitutions at Y42, S185, E187, L189 and L218 inactivated the function of TatC (Fig. 3, top panel); however, they did not show dominant negative activity when produced in a *tat* ^+^ strain background (Fig. 3, bottom panel). Likewise, all three of the TatBC fusions we tested were also able to support Tat transport activity (Fig. 3, top panel) – again this was not surprising as a TatBC fusion protein has been constructed previously that retains Tat function (Bolhuis *et al*., 2001). However, we did note that when these fusions were produced in the *tat* ^+^ strain, the strain failed to grow anaerobically with TMAO as sole electron acceptor, indicating that they had some degree of dominant negative activity, although a reasonable level of growth was seen on SDS‐containing medium. When the stop codon mutations (or in some cases co‐occurring point mutations) were additionally introduced into these fusion constructs and the plasmids introduced into the *tat* ^+^ strain MC4100, growth on SDS was severely affected. We conclude that the TatBC fusions display a partial dominant negative phenotype that is compounded if TatC is C‐terminally truncated.

### The dominant negative TatC variants self‐interact and still bind TatB and TatA

**Figure 4:**
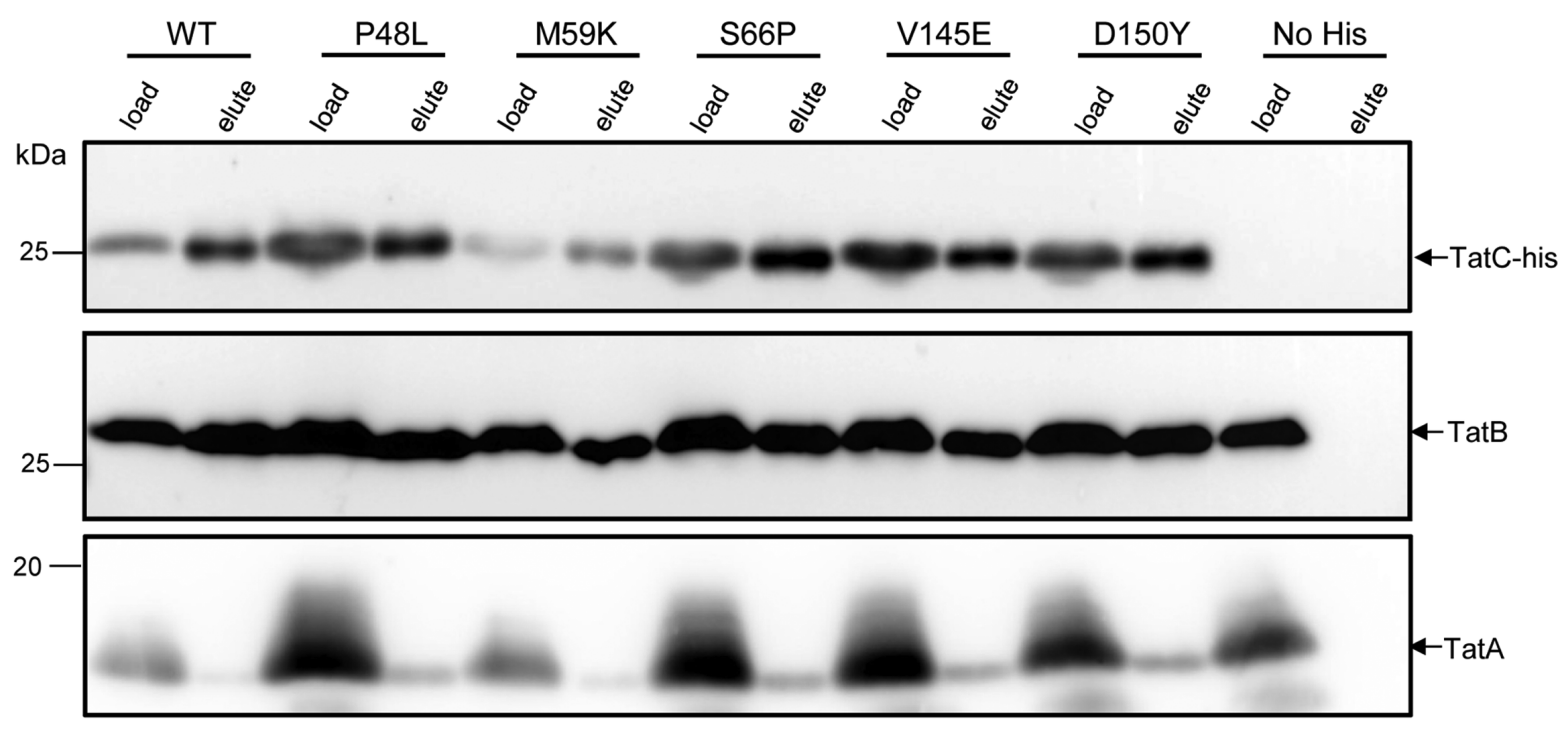
Dominant negative point substituted variants of TatC interact with TatB and TatA. Crude membrane fractions of the *E* *. coli* strain DADE (Δ*tat* *ABCD* Δ*tatE*) harbouring pREP4 (Zamenhof and Villarejo, 1972) and over‐producing TatA, TatB and hexa‐histidine‐tagged wild‐type or amino‐acid substituted TatC, as indicated were solubilised with digitionin and the TatC‐his protein purified using nickel‐charged beads as described in Experimental Procedures. In each case a sample that was loaded onto the beads (load) along with the sample that was eluted from the beads (elute) were separated by SDS–PAGE (12% acrylamide), electroblotted and immunoreactive bands were detected with either anti‐his, anti‐TatB or anti‐TatA antisera. As a control, solubilised membranes from strain DADE/pREP4 overproducing TatA, TatB and a non‐tagged variant of TatC (lanes labelled No his) were used to show that there was no unspecific binding of TatA and TatB to the beads. Five microlitres of sample was loaded in each lane.

In order to characterise the dominant negative single amino‐acid variants of TatC, we first tested whether TatC P48L, TatC M59K, TatC S66P, TatC V145E and TatC D150Y retained interactions with TatB and TatA. To facilitate this, the wild‐type and variant TatC proteins were supplied with a C‐terminal hexahistidine tag and co‐overproduced with TatA and TatB from the pUNITAT2 plasmid (McDevitt *et al*., 2005). Membrane proteins were solubilised with digitonin and TatC‐his‐containing complexes isolated using nickel beads. It can be seen in Fig. 4 that TatB along with low levels of TatA specifically eluted with TatC‐his from the beads, even when dominant negative substitutions were present in TatC. Therefore the interaction of TatA and TatB partner proteins is not grossly disrupted by the presence of dominant negative amino‐acid substitutions.

**Figure 5:**
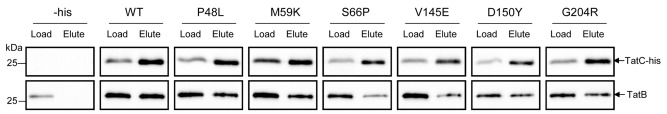
Dominant negative point substituted variants of TatC do not prevent his‐tagged wild‐type TatC from interacting with TatB. Crude membrane fractions of *E* *. coli* strain MC75CH (as MC4100, *tat* *C* *_his_*) harbouring pTTC1 and over‐producing TatA, TatB and wild‐type or amino‐acid substituted TatC, from pTAT1d as indicated, were solubilised with digitonin and the chromosomally encoded TatC‐his protein from the chromosome of strain MC75CH was purified using nickel‐charged beads as described in Experimental Procedures. In each case, a sample that was loaded onto the beads (load) along with the sample that was eluted from the beads (elute) were separated by SDS–PAGE (12% acrylamide), electroblotted and immunoreactive bands were detected with either anti‐His or anti‐TatB. As a control, solubilised membranes from strain MC4100[pTTC1] overproducing TatA, TatB and a non‐tagged variant of TatC (lane labelled ‐his) were used to show that there was no unspecific binding of TatB to the beads.

A potential explanation for the effect of the dominant negative TatC substitutions that would not need to invoke TatC oligomerisation is that the variants have additional high affinity TatB binding sites (for example, by converting one or more TatA binding sites to TatB binding), which compete the available TatB protein away from wild‐type TatC. To test this possibility, we utilised strain MC75CH that codes for a C‐terminally his‐tagged TatC at the native chromosomal locus. We co‐produced TatA and TatB along with untagged wild‐type or dominant negative P48L, M59K, S66P, V145E or D150Y TatC variants. TatC variant G204R was also analysed as a negative control. It can be seen (Fig. 5) that TatB co‐purifies with the his‐tagged TatC both in the presence of co‐produced wild‐type TatC and in the presence of the dominant negative TatC variants. We conclude that there is no evidence that the dominant negative substitutions titrate TatB away from wild‐type TatC.

**Figure 6:**
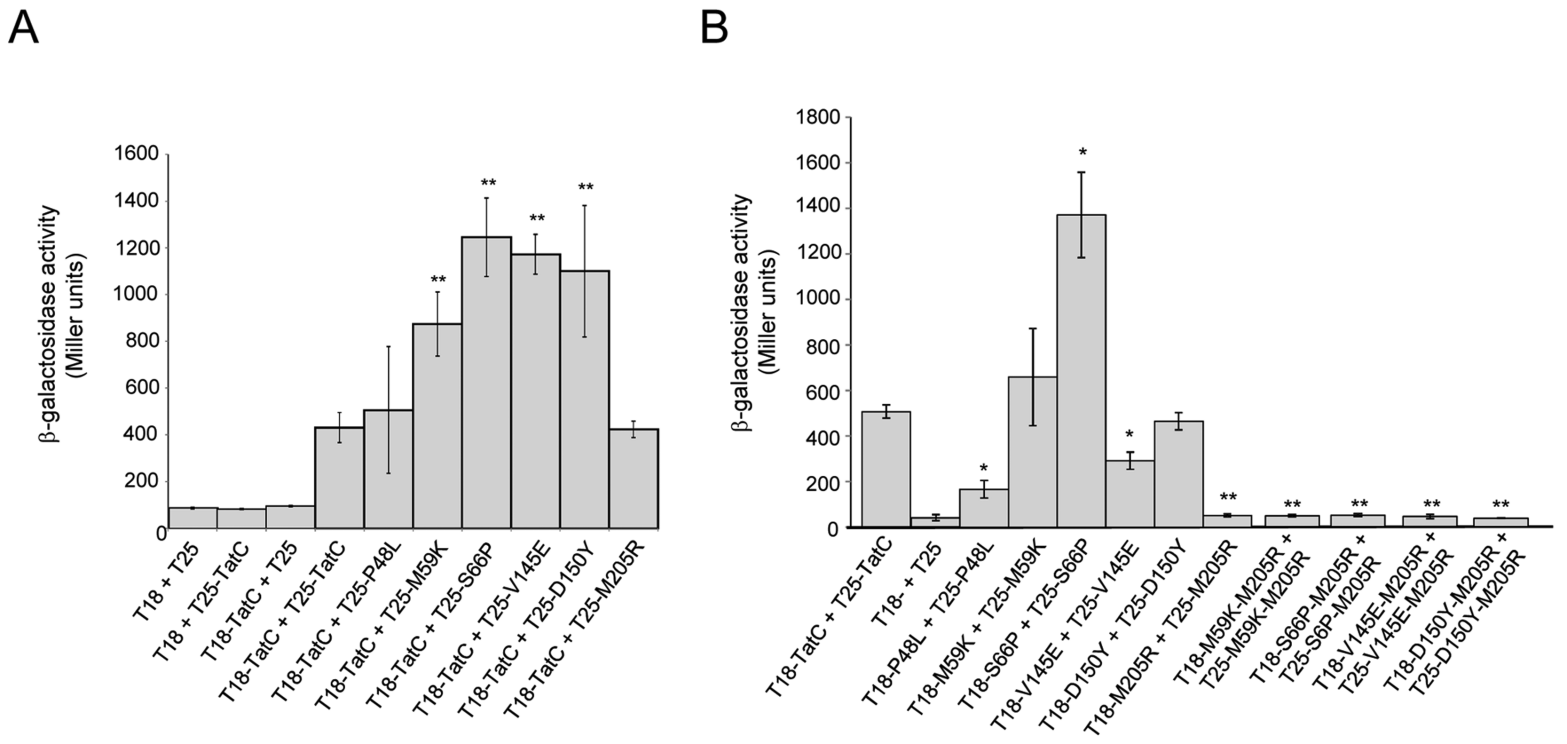
Bacterial two hybrid analysis of TatC–TatC interaction. A and B. Interactions between the indicated variants of TatC fused to either the T18 or T25 fragments of *Bordetella pertussis* adenylate cyclase, as indicated. Error bars represent the standard error of the mean (*n* = 6; two technical replicates of three biological replicates). Significance was assessed using Student's *t*‐test, where asterisk signifies *P* < 0.005 and double asterisks signifies *P* < 0.001 relative to the value for T18‐TatC + T25‐TatC.

The bacterial two hybrid system developed by Karimova *et al*. (1998) can be applied to assess interactions between membrane proteins (e.g. Karimova *et al*., 2005). When one TatC molecule was fused to the N‐terminus of the adenylate cyclase T18 fragment and a second to the C‐terminus of the T25 fragment, adenylate cyclase activity was reconstituted, and strong β‐galactosidase activity could be measured (Fig. 6A). It should be noted that this probably represents a direct TatC–TatC interaction rather than being mediated through TatB because although the BTH101 strain used for these experiments encodes chromosomal *tatABC*/*tatE*, the TatC fusions are produced in multicopy under the control of the strong *lacUV5* promoter (Karimova *et al*., 1998). We next assessed using this method whether the five amino‐acid substituted dominant negative TatC variants produced as T25 fusion proteins could interact with the wild‐type TatC that was produced as a fusion to T18. Figure 6A indicates that indeed interaction between the wild‐type and dominant negative variants of TatC could be detected. For T25 fusions of four of the five dominant negative TatC variants, M59K, S66P, V145E and D150Y, the level of β‐galactosidase activity measured in the presence of T18 wild‐type TatC was significantly higher than that measured for the T25 wild‐type TatC fusion.

We also assessed whether each of the dominant negative TatC variants could self‐interact using this approach, and indeed Fig. 6B shows that self‐interaction could be seen for each of the variants. The S66P variant gave particularly high levels of β‐galactosidase activity, significantly above that seen for the wild‐type TatC self‐interaction, whereas two of the variants, P48L and V145E, gave β‐galactosidase activity levels lower than the wild type, but still significantly higher than the negative control.

### The dominant negative TatC proteins can interact with substrate proteins but are unable to trigger TatA polymerisation

**Figure 7:**
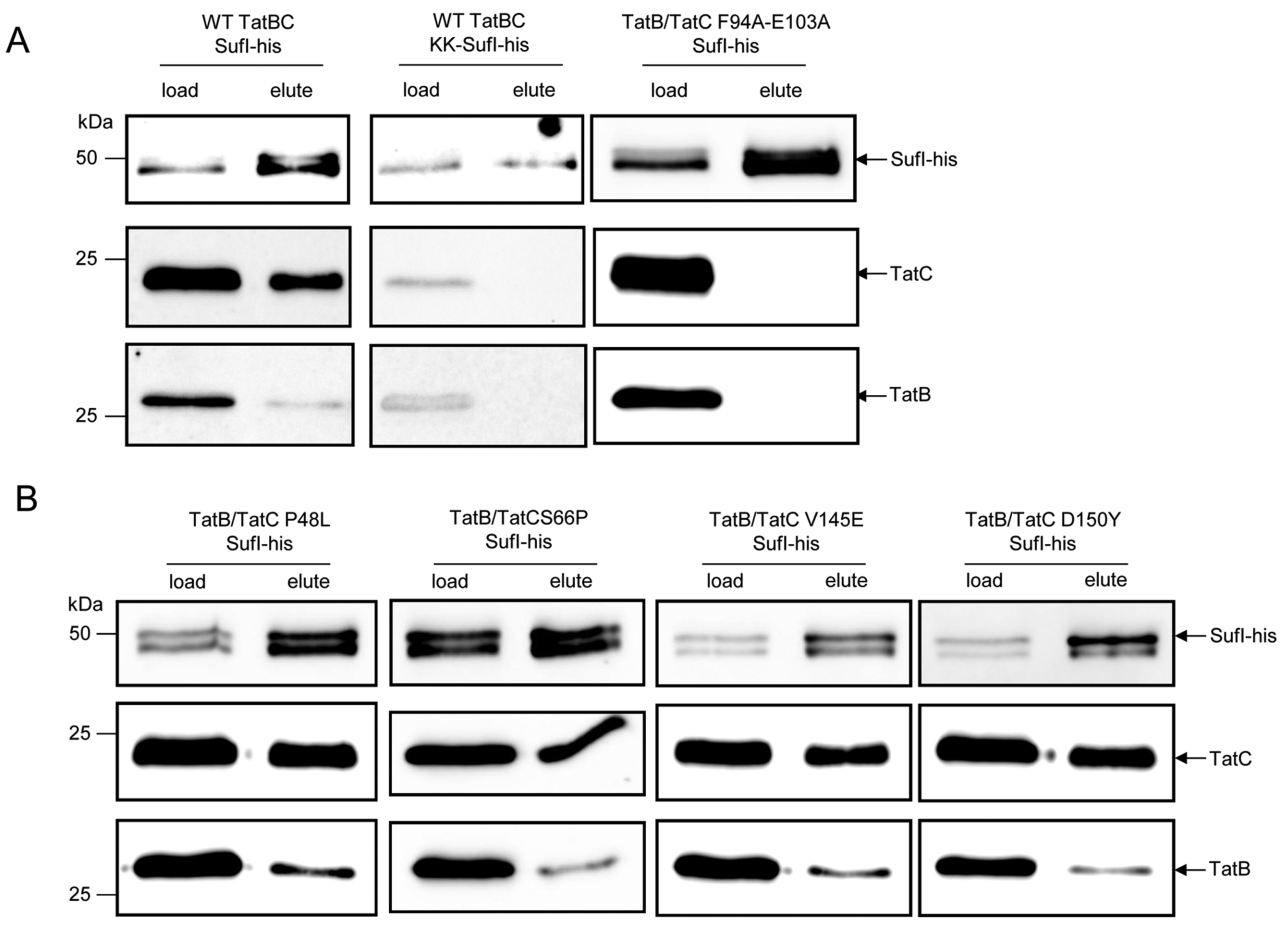
Dominant negative TatC proteins can be co‐purified with the Tat substrate, SufI. Membrane fractions were prepared from strain DADE (Δ*tat* *ABCD*, Δ*tat* *E*) harbouring pREP4 and either pFAT75ΔASufIhisKK or pFAT75ΔASufIhis coding for wild‐type or variant TatC (along with wild‐type TatB and his‐tagged SufI) as indicated, and resuspended in buffer to give an equivalent protein concentration. Following solubilisation with 1% digitonin, detergent‐extracted proteins were loaded onto nickel‐charged resin, washed with buffer containing 50 mM imidazole and finally eluted from the column in the presence of 10 mM EDTA. Samples (5 μl) of the load and eluate fractions were separated by SDS–PAGE (12% acrylamide) and analysed by immunoblotting with anti‐his, anti‐TatC and anti‐TatB antibodies.

To ascertain which step/s in the Tat transport pathway were blocked by the TatC dominant negative amino‐acid substitutions, we next examined whether the TatC substitutions P48L, S66P, V145E or D150Y affected interaction of substrate proteins with TatBC. It has been shown previously that SufI can bind tightly to membranes containing TatBC (Bageshwar *et al*., 2009) and that a ternary complex can be purified following detergent extraction (Tarry *et al*., 2009). We therefore designed a plasmid that would allow co‐production of TatB and TatC with a his‐tagged SufI precursor, isolated membrane fractions from cells containing this plasmid, solubilised these with digitonin and purified SufI‐his using Ni‐charged beads. Figure 7A shows that following elution of bound SufI from the beads, wild‐type TatB and TatC could be co‐eluted, indicative of complex formation. Further control experiments (Fig. 7A) showed that no TatB or TatC could be recovered when the conserved twin‐arginine motif of the SufI signal peptide was mutated to twin lysine (a substitution which is known to abolish targeting of SufI to the Tat pathway; Stanley *et al*., 2000), nor when the TatC protein harboured the F94A, E103A double substitution that is known to block signal peptide binding (Holzapfel *et al*., 2007; Rollauer *et al*., 2012; Alcock *et al*., 2013). However, it can clearly be seen (Fig. 7B) that the dominant negative TatC substitutions do not block substrate binding, since in each case, the variant TatC proteins could be co‐purified, along with TatB, by interaction with his‐tagged SufI.

**Figure 8:**
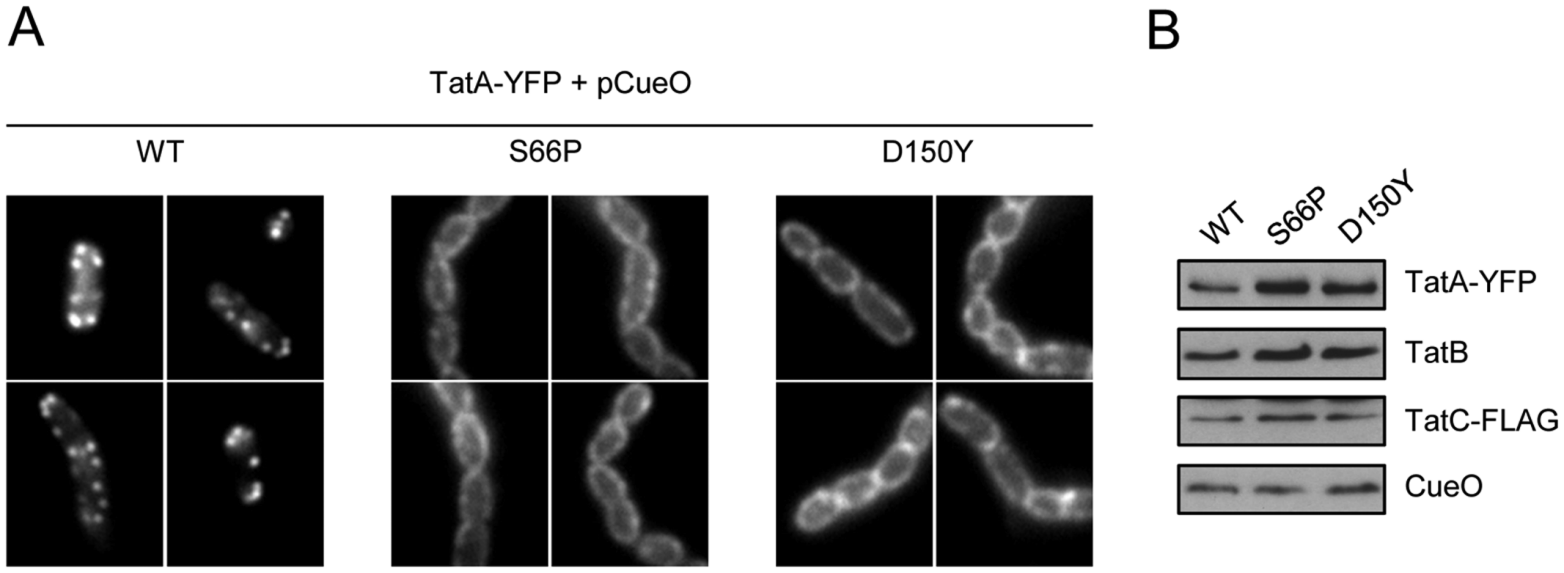
Substrate‐induced assembly of TatA‐YFP is blocked by the TatC S66P and D150Y mutations. The Tat substrate CueO was overproduced from plasmid pQE80‐CueO (Leake *et al*., 2008) in strain MΔABC‐A λAry p101C*TatBCflag encoding TatB along with the wild‐type or dominant negative TatC variants as indicated, with a C‐terminal FLAG tag on TatC. A. Fluorescence micrographs showing the distribution of TatA‐YFP following overproduction of CueO for the indicated TatC variants. B. Isolated membranes of the same strains used for microscopy analysed by Western blot with antibodies against TatA, TatB or FLAG, or whole cell extracts were analysed similarly with antibodies against CueO. Eighteen micrograms of membranes were loaded per lane. CueO antibodies were raised in rabbits against the mature protein, and affinity purified.

Once a substrate is bound to the TatBC complex, the next step of Tat transport is the recruitment of TatA, in multiple copies, to assemble the active translocation site (Mori and Cline, 2002; Alami *et al*., 2003; Alcock *et al*., 2013). This can be visualised in intact *E. coli* cells using a chromosomally encoded TatA–YFP protein. This fusion protein, in the presence of TatBC and low levels of the TatA paralogue TatE, supports full physiological Tat transport activity (Alcock *et al*., 2013). When the Tat substrate protein CueO is overproduced in this strain background, bright foci of TatA–YFP can be observed by fluorescence microscopy (Alcock *et al*., 2013; Fig. 8A). However, when either the S66P or D150Y dominant negative substitutions were present in TatC (which also carried a C‐terminal FLAG epitope in these experiments), TatA–YFP showed only dispersed fluorescence even in the presence of overproduced CueO (Fig. 8A). Western blotting analysis confirmed that the Tat components and CueO were present at similar levels in all of the strains analysed (Fig. 8B). We conclude that although the TatC dominant negative variants are able to interact with substrate proteins, at least two of them are unable to trigger the substrate‐induced recruitment of TatA.

### The TatBC complex does not fully assemble in the presence of dominant TatC variants

The experiments undertaken so far have established that the dominant negative amino‐acid substitutions of TatC do not prevent TatC self‐interaction, as assessed by the bacterial two hybrid assay, nor do they prevent association with TatB or substrate protein. To determine whether they result in any gross conformational or oligomer state changes in the TatBC complex, BN‐PAGE was performed on digitonin‐solubilised membranes from strain DADE (Δ*tatABCD*, Δ*tatE*) producing the TatABC proteins from the medium copy number pTAT1d plasmid.

**Figure 9:**
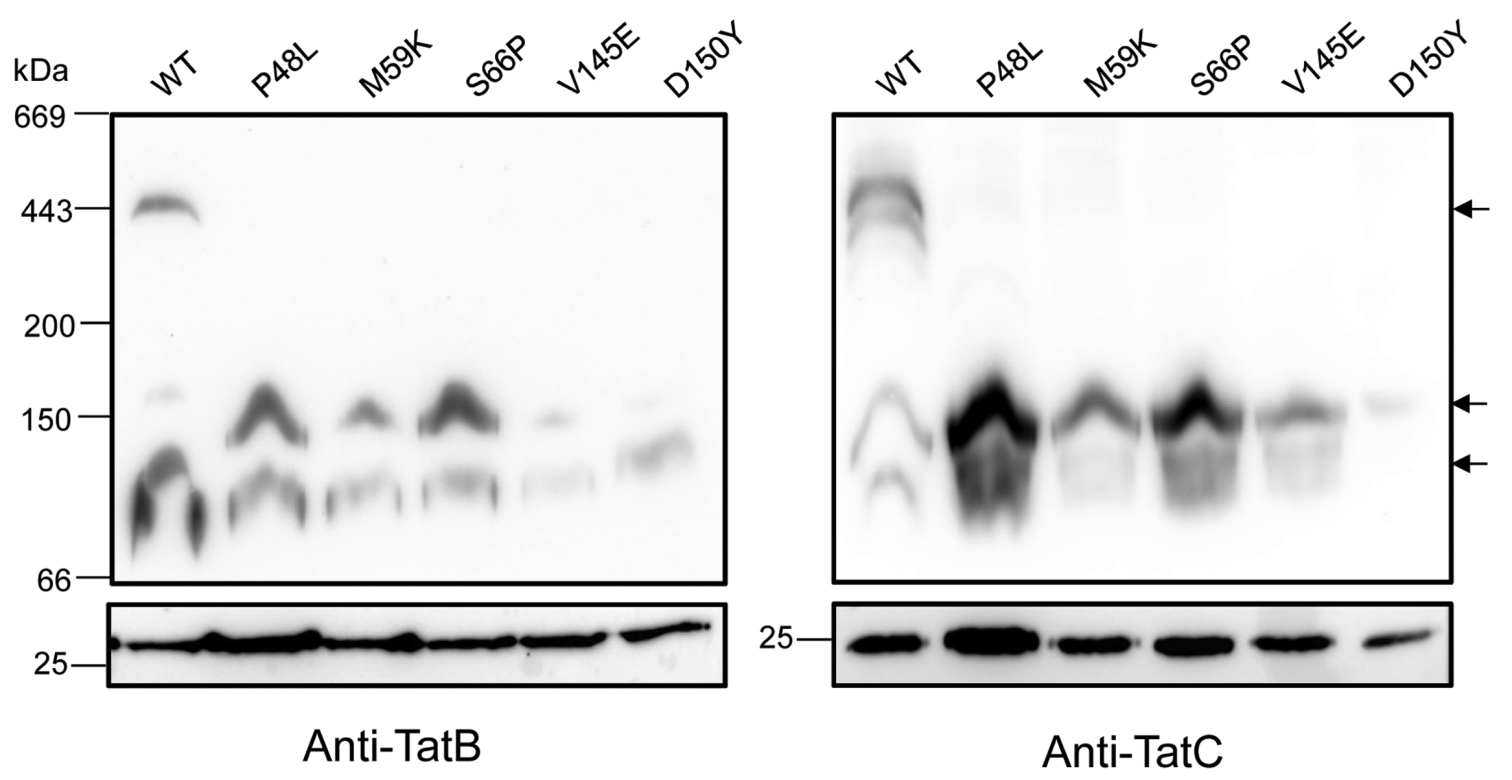
Blue native (BN)‐PAGE analysis of TatBC complexes containing dominant negative TatC substitutions. Crude membrane fractions of the *E* *. coli* strain DADE (Δ*tat* *ABCD* Δ*tat* *E*) overproducing wild‐type or P48L, M59K, S66P, V145E or D150Y amino‐acid substituted TatC along with TatA and TatB from the pTAT1d plasmid were solubilised using 2% digitonin and samples (approximately 200 μg protein) were analysed by BN‐PAGE. The arrows to the right indicate TatBC‐containing complexes. The bottom panels show SDS–PAGE analysis of the same solubilised samples.

Figure 9 shows that when the wild‐type allele of *tatC* was present on the plasmid, the major band cross‐reacting with both TatB and TatC antisera migrates at just over 440 kDa. Other bands at lower molecular weight are also visible and have been reported previously (e.g. Behrendt *et al*., 2007; Orriss *et al*., 2007; Richter and Bruser, 2005). However, when any of the five dominant negative amino‐acid substitutions were introduced into TatC, the 440 kDa TatBC complex was no longer detected, and instead smaller complexes that cross‐reacted with both the TatB and TatC antibodies accumulated migrating at approximately 100 and 150 kDa were observed. These small complexes detected in the presence of dominant negative TatC variants migrated at the same size as the minor lower molecular weight bands detected for the wild‐type TatC‐containing complexes. We therefore conclude that the dominant negative amino‐acid substitutions either greatly destabilise the 440 kDa TatBC complex or prevent its assembly from TatBC subcomplexes.

### A TatC self‐interaction site at the periplasmic cap is maintained in the presence of dominant negative substitutions

**Figure 10:**
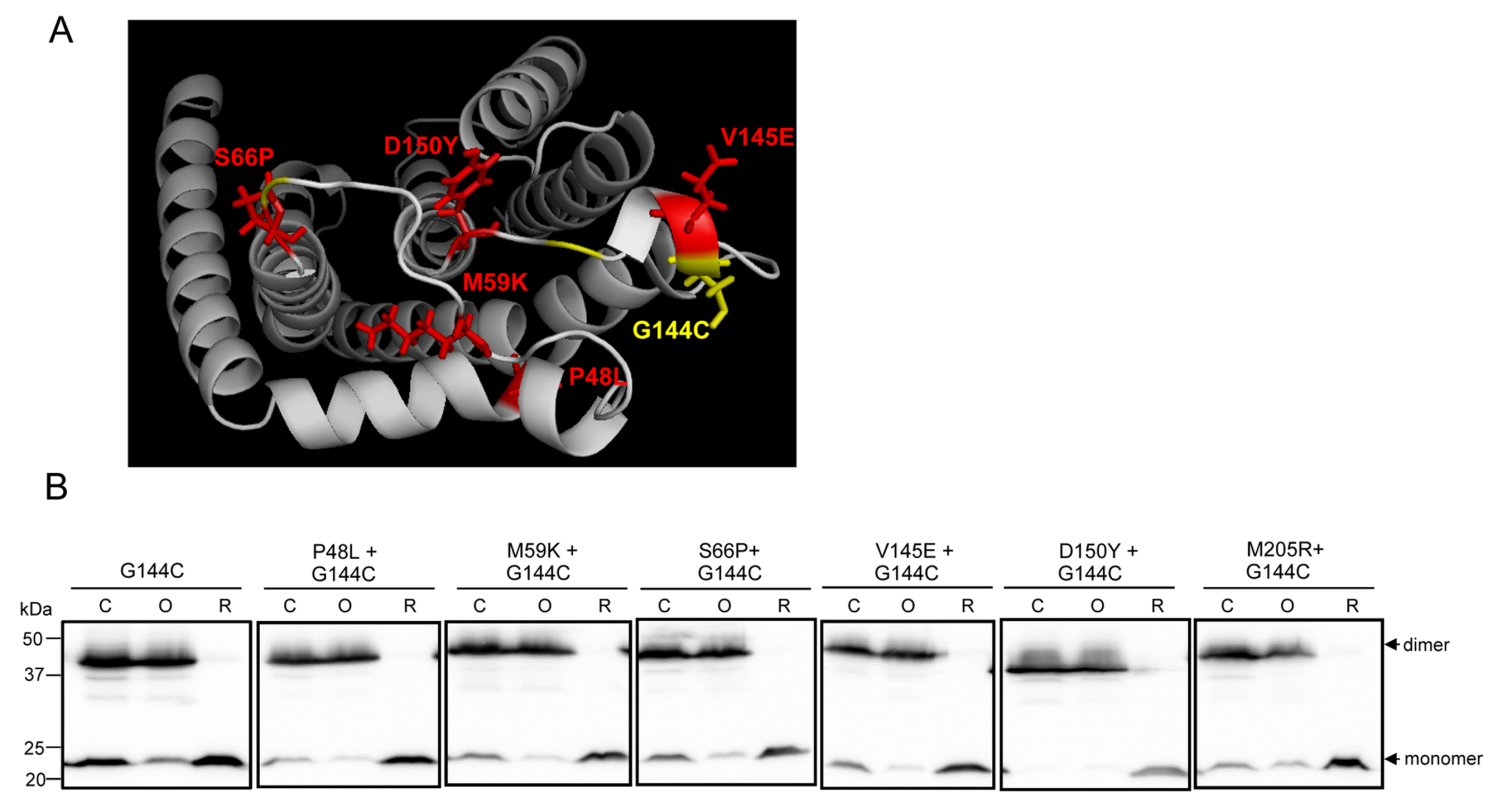
A TatC self‐crosslink in the periplasmic cap of TatC is not affected by dominant negative substitutions. A. Periplasmic view of a homology model of *E. coli* TatC showing positions of the P48L, M59K, S66P, V145E and D150Y dominant negative amino‐acid substitutions (red) and G144C substitution (yellow) used for disulphide crosslinking analysis. B. Intact cell suspensions (50 ml of culture of OD_600nm_ = 0.15) of strain DADE (Δ*tatABCD* Δ*tatE*) co‐producing TatA, TatB and the single cysteine variant G144C of TatC together with additional TatC substitutions, as indicated, from pTat101 were left untreated (control sample; C), or subjected to either oxidising (O) or reducing (R) conditions as described in Experimental Procedures. Samples (50 μg of membrane protein) were resolved by SDS–PAGE (12% acrylamide), and TatC monomers and dimers visualised by western blotting.

Crosslinking experiments have implicated the periplasmic cap of TatC as a likely site of TatC self‐interaction (Punginelli *et al*., 2007; Zoufaly *et al*., 2012). Given that the dominant negative amino‐acid substitutions are almost exclusively associated with this region, it is possible that they may perturb the ability of TatC to interact through the cap and that this might account for the smaller TatBC complexes seen on BN‐PAGE. It was noted previously that when residue G144 in the second periplasmic loop region was mutated to cysteine, an almost quantitative self‐crosslink was observed when the Tat proteins were overproduced (Punginelli *et al*., 2007). To probe this further, we expressed the G144C variant of TatC at close to native levels of expression from plasmid pTat101 (Kneuper *et al*., 2012), alongside TatA and TatB, and we developed a method for disulphide crosslinking in living cells. Figure 10B shows that *in vivo*, there is extensive self‐crosslinking of TatC through the introduced Cys residue, even in the absence of exogenous oxidising agent, strongly suggesting that there is a *bona fide* TatC contact site close to this position.

We next tested whether the G144C crosslink could still be detected when dominant negative substitutions were also introduced into TatC. The position of the G144C residue relative to each of the P48L, M59K, S66P, V145E or D150Y dominant negative amino‐acid substitutions is shown in Fig. 10A. Strikingly, a very strong disulphide bond at G144C was able to form despite introduction of any of the dominant negative substitutions (Fig. 10B). We conclude that this TatC self‐interaction site is maintained in the dominant negative TatC variants.

### A substrate‐induced crosslink at TatC M205C is blocked by the TatC dominant negative substitutions

Previously, a second potential TatC self‐interaction site was found in transmembrane helix 5 of TatC, at M205C (Kneuper *et al*., 2012; Rollauer *et al*., 2012). This site is close to the TatB binding site, and the M205R inactivating substitution of TatC can be suppressed by substitutions in the transmembrane helix of TatB. To explore potential TatC dimerisation mediated through this region of TatC, we first introduced the M205R substitution into TatC and investigated whether the variant proteins could interact in the bacterial two‐hybrid assay. Figure 6A shows that when this point substitution was present in TatC fused to T25, it was able to interact with a wild‐type copy of TatC fused to T18. However, when the TatC fused to T18 and T25 both carried the M205R substitution, no dimerisation was detected (Fig. 6B). Introduction of any of the dominant negative amino‐acid substitutions M59K, S66P, V145E or D150Y alongside TatC M205R did not restore TatC dimerisation (Fig. 6B). Therefore, we conclude that in the bacterial two hybrid assay, TatC self‐interaction is likely mediated by a dimer interface that encompasses transmembrane helix 5.

**Figure 11:**
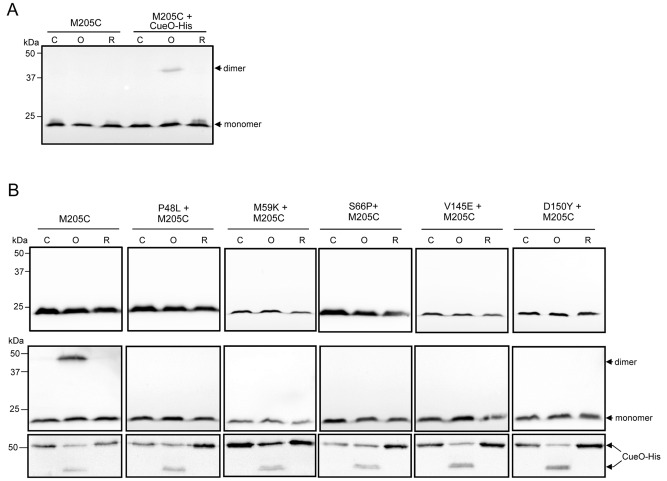
A substrate‐induced TatC self‐crosslink in transmembrane helix 5 of TatC. A. Whole cells of strain DADE (Δ*tatABCD* Δ*tatE*) harbouring pTat101 co‐producing TatA, TatB and the single cysteine variant M205C of TatC, alone or with additional plasmid pQE80‐CueO were cultured as described in Experimental Procedures and subjected to oxidising (O) or reducing (R) conditions, or left untreated (control; C). Samples (50 μg of membrane protein) were resolved by SDS–PAGE (12% acrylamide), and TatC monomers and dimers visualised by western blotting using an anti‐TatC antibody. B. Cells of the same strain harbouring pTat101 co‐producing TatA, TatB and the single cysteine variant M205C of TatC together with the indicated additional TatC substitutions alone (top panel) or with pQE80‐CueO (middle and bottom panels) were treated as in part A and samples (50 μg of membrane protein) were resolved by SDS–PAGE (12% acrylamide), and blotted with anti‐TatC (middle panel) or anti‐his antibody (bottom panel). Note that the fast migrating band detected on the anti‐his blot for the oxidised sample most probably represents a crosslinked form of CueO containing an intramolecular disulphide.

Disulphide crosslinking studies of TatC M205C have shown that the residue exhibits unusual behaviour because it can not only self‐crosslink but can also crosslink to cysteine residues introduced into the transmembrane helix of TatB (Kneuper *et al*., 2012; Rollauer *et al*., 2012). However, as before, these experiments were undertaken *in vitro*, with membranes containing high levels of Tat components. To examine this potential TatC interaction site under more native conditions, we assessed whether a disulphide bond could be formed between two TatC M205C variants *in vivo* when the Tat components were produced at close to native level. As shown in Fig. 11A, *in vivo* there was no detectable TatC dimerisation unless the Tat substrate protein, CueO, was co‐overproduced, in which case a TatC dimer band was clearly visible under oxidising conditions. We conclude that M205C residues from neighbouring TatC proteins must come into close proximity when substrates bind to the TatBC complex.

To determine whether any of the dominant negative substitutions P48L, M59K, S66P, V145E or D150Y altered the crosslinking behaviour of M205C, the same *in vivo* crosslinking experiments were carried out with TatC M205C that additionally harboured a single dominant negative substitution. Figure 11B shows that no disulphide crosslink is able to form between M205C residues when any of the five dominant negative substitutions are present, regardless of the presence of overproduced CueO. It therefore appears that these substitutions prevent the function of wild‐type TatC by blocking the formation of a transient TatC dimer that forms in response to substrate binding during Tat protein translocation.

## Discussion

In this study we have sought to test whether oligomerisation of TatC is a functional requirement of the Tat pathway by undertaking a genetic screen to identify mutant alleles of *tatC* that interfere with the function of the wild‐type protein. Our success in isolating such mutants strongly suggests that co‐operation between at least two protomers of TatC is an essential step in the protein translocation pathway.

The dominant negative mutant alleles we isolated fell into two classes. One class encoded TatBC fusion proteins that frequently contained stop codon mutations within *tatC*, leading to C‐terminal truncations. Dissecting out these mutations revealed that it was necessary for the truncated TatC variants to be fused to TatB in order for them to have dominant negative activity. This can be rationalised if the truncated TatC proteins alone are unable to stably interact with the TatBC complex, but the TatB portion of the fusion integrates into the complex, bringing the truncated TatCs into close proximity with the wild‐type protein. Since these mutant alleles are likely to grossly perturb the structure and stability of dominant negative variants, we did not study them further.

The second class of dominant negative mutant alleles we isolated encoded one or more amino‐acid substitutions in TatC that predominantly localised to the first and second periplasmic loops, which together form the periplasmic cap region of the protein. These substitutions did not prevent TatC from interacting with TatB or TatA, but BN‐PAGE analysis showed that TatBC complexes containing these dominant negative amino‐acid substitutions did not assemble into the large (approximately 440 kDa) hetero‐oligomer formed from TatB and wild‐type TatC. Instead, smaller complexes, most likely subcomplexes of the 440 kDa complex, accumulated. Other substitutions in these loop regions have previously been noted to affect assembly of the TatBC complex (Barrett *et al*., 2005; Behrendt and Bruser, 2014), and of the thylakoid Hcf106‐cpTatC receptor complex (Ma and Cline, 2013). It therefore seems likely that at least one of the TatC contact sites is mediated through the cap region of TatC.

However, the effect of these substitutions is not as simple as abolishing TatC interaction via the cap. Disulphide crosslinking of residue G144C, which is present in the cap region, was not affected by the introduction of the single dominant negative substitutions, nor were disulphide crosslinks at A65C or G148C (not shown). Conformational change at the cap region is likely to be important during the different steps of protein translocation, for example the G144C variant of TatC, which is extensively disulphide‐locked *in vivo*, is associated with low transport activity (Punginelli *et al*., 2007). Furthermore, it has also been noted that a disulphide crosslink at position L126 in the cap region of thylakoid TatC decreased in intensity in the presence of a Tat substrate (Aldridge *et al*., 2014). The dominant negative point substitutions could potentially exert their effect by stabilising one particular cap conformation and preventing progression through the transport cycle.

Transmembrane helix 5 of TatC is a short, sharply kinked helix that is too short to fully span the lipid bilayer (Rollauer *et al*., 2012; Ramasamy *et al*., 2013). Several studies have pinpointed the C‐terminal (periplasmic) end of this helix as playing a critical role in Tat transport. For example residues in this helix interact with TatB (Kneuper *et al*., 2012; Rollauer *et al*., 2012), and in the chloroplast Tat system, this helix of cpTatC has been shown to interact with the TatA orthologue, Tha4, and with substrate signal peptides (Aldridge *et al*., 2014). In all of these studies, TatC self‐contacts were also detected at specific positions within this helix. Here we noted that this helix also forms part of a TatC dimer interface detected using the bacterial two‐hybrid system. When the Tat‐inactivating M205R substitution, which falls close to the C‐terminal end of helix 5, was introduced into TatC, no dimerisation was detected, supporting the inference from crosslinking that this is a site of self‐interaction. However, when we undertook disulphide crosslinking experiments *in vivo*, we did not detect disulphide formation at TatC M205C unless we overproduced a substrate protein. We interpret these findings to suggest that dimerisation at M205C is transient and occurs at one of the stages in the Tat transport cycle, most likely following engagement of a signal peptide with the TatBC complex. We suggest that in the resting state, the M205C residues from adjacent TatC proteins are held apart, for example by the presence of an intervening TatB or TatA molecule, and that there is conformational rearrangement upon signal peptide binding to bring M205C residues into close proximity. This would also explain why the M205 dimer interface is detected in the bacterial two hybrid system, since in these experiments, the TatC fusions are overproduced in the presence of only native levels of the TatB or TatA partner proteins that may normally mask this binding site.

Aldridge *et al*. (2014) have reported a pea chloroplast TatC–TatC disulphide crosslink at a position (V270) corresponding to L206 in *E. coli* TatC and thus directly adjacent to the M205C crosslink analysed here. The crosslinking at this position in the chloroplast TatC displays interesting behaviour since it self‐crosslinks in the absence of Tha4 (the chloroplast equivalent of TatA) but in the presence of Tha4 preferentially crosslinks to Tha4 instead of a neighbouring TatC. However, in the additional presence of substrate, the TatC homodimeric crosslink is not restored, in contrast to the behaviour of the *E. coli* TatC M205C crosslink, which is generated only when substrate is overproduced. Instead, the cpTatC‐Tha4 crosslink diminishes and there is a crosslink between cpTatC V270C and the substrate signal peptide (Aldridge *et al*., 2014). It is likely that these differences in crosslinking behaviour reflect the different positions on transmembrane helix 5 that are probably occupied by these two residues, with *E. coli* TatC L206/cpTatCV270 predicted to point into the concave face of TatC, whereas M205 is predicted to point outwards. None‐the‐less, the data presented here and in the study of Aldridge *et al*. (2014) consistently indicate that there is change in the dimerisation interface at the periplasmic (luminal) end of TatC helix 5 with differing states of the Tat translocase. That the dominant negative variants of TatC are unable to generate the homodimeric crosslink at M205C in the presence of substrate, despite retaining the ability to interact with substrate, adds further support to the notion that these variants are unable to trigger events, including TatA recruitment and potentially TatBC reorganisation, that might occur following binding of substrates.

## Experimental procedures

### Bacterial strains, plasmids and growth conditions

Strains MC4100 [F^−^, [*araD139*]*_B/r_*, Δ(*argF‐lac*)*U169*, *λ* ^−^, *e14*‐, *flhD5301*, Δ(*fruK‐yeiR*)*725*(*fruA25*), *relA1*, *rpsL150*(Str^R^), *rbsR22*, Δ(*fimB‐fimE*)*632*(*::IS1*), *deoC1* – (Casadaban and Cohen, 1979)] and DADE [As MC4100, Δ*tatABCD*, Δ*tatE* – (Wexler *et al*., 2000)] were used in this study. Strain MC75CH is derived from MC4100 but encodes a hexahistidine tag coding sequence at the 3′ end of *tatC* and has been described previously (McDevitt *et al*., 2006). Bacterial two hybrid analysis was carried out in strain BTH101 [F^−^ *cya‐99*, *araD139*, *galE15*, *galK16*, *rpsL1* (Str^r^), *hsdR2*, *mcrA1*, *mcrB1* (Karimova *et al*., 2000)]. Strain MΔABC‐A* λAry, which is deleted for *tatABC* at the native chromosomal location and harbours a *tatA‐yfp* allele integrated at the *attB* site, was used for fluorescent microscopy experiments and has been described previously (Alcock *et al*., 2013).

To produce TatC as a fusion protein to the N‐terminus of the T18 fragment of adenylate cyclase of *Bordetella pertussis*, *tatC* was amplified with oligonucleotides pUT18TatCforXba and pUT18TatCrevBam (Table S2), using MC4100 chromosomal DNA as template. The resultant product was digested with *Xba*I and *Bam*HI and cloned into pUT18 (Karimova *et al*., 2001) to give plasmid pUT18‐TatC. Similarly, TatC was produced as a C‐terminal fusion to the *B. pertussis* adenylate cyclase T25 fragment following amplification with oligonucleotides pT25TatCforBam and pT25TatCrevKpn, digestion with *Bam*HI and *Kpn*I and cloning into pT25 (Karimova *et al*., 1998). Plasmid pTAT1d contains a modified *tatABC* operon expressed from the native *tatA* promoter in plasmid pT7.5 and pTTC1 encodes CAT fused to the TorA signal peptide, also driven from the *tatA* promoter – both have been described previously (Maldonado *et al*., 2011b). For TatC purification of plasmid‐encoded his‐tagged TatC variants, these were co‐produced with TatA and TatB from plasmid pUNITAT2 (McDevitt *et al*., 2005). To co‐produce TatB and TatC variants along with a C‐terminally his‐tagged SufI protein, plasmid pFAT75ΔA (Tarry *et al*., 2009) was modified as follows. First the 3′ part of the *tatC* gene on this plasmid was modified by introduction of the *tatC* stop codon, followed by *Apa*I restriction site. This was achieved by amplifying a portion of *tatC* using oligonucleotides tatC6741 and tatCstopApa (Table S2), digestion with *Eco*47III and *Bgl*II and cloning into pFAT75ΔA cut with same enzymes to give pFAT75ΔAApa. Next, the *sufI* gene was amplified with oligonucleotides SufIstartApa and SufIBglII (Table S2), digested with *Apa*I and *Bgl*II fragments and cloned into similarly digested pFAT75ΔAApa to give pFAT75ΔASufIhis. In parallel, a *sufI* gene encoding for a twin lysine substitution of the twin‐arginine motif of the SufI signal peptide was also amplified using pT7.5 SufIKK as a template (Stanley *et al*., 2000) and similarly cloned to give pFAT75ΔASufIhisKK. For *in vivo* disulphide cross‐linking experiments, TatC variants were co‐produced alongside TatA and TatB from plasmid pTat101 or TatB only from plasmid pTatBC101, both constructed with the vector backbone pTH19kR and used for low‐copy expression (Kneuper *et al*., 2012; Alcock *et al*., 2013). For TatC detection following production from very low copy number plasmids, a FLAG epitope coding sequence was introduced at the 3′ end of *tatC* on plasmid p101C*TatBC using primers p101C*BCflag_F and p101C*BCflag_R (Table S2). A complete list of all plasmids used and constructed in this study is given in Table S1.

To introduce nucleotide exchanges into *tatC*, the Quickchange site‐directed mutagenesis protocol (Stratagene) was used – all primers used for site‐directed mutagenesis and other aspects of this study are listed in Table S2. Amplified products were digested with *Dpn*I and subsequently used to transform *E. coli* strain DH5α [*F* ^−^ φ*80dlacZM15* (*lacZYA‐argF*)*U169 deoR recA1 endA1 hsdR17*(*r k* ^−^ *, mk^+^*) *phoA supE44 thi‐1 gyrA96 relA1* λ^−^]. Modified plasmids were isolated and the introduction of the selected mutations was confirmed by sequencing of the whole *tatABC* operon.

Phenotypic growth screening in the presence of chloramphenicol was undertaken as described previously, with the exception that 200 μg ml^−1^ chloramphenicol was used (Maldonado *et al*., 2011b; Kneuper *et al*., 2012). Growth of strains on 2% SDS, or anaerobically with glycerol and TMAO, was tested as described previously (Buchanan *et al*., 2002; Palmer *et al*., 2010). For direct comparison of the activity of Tat systems harbouring variants of TatC, 5 ml liquid cultures was grown in Luria–Bertani (LB) medium containing the appropriate antibiotics with shaking at 37°C for 8 hours and diluted in phosphate‐buffered saline to give a final OD_600_ of 0.0001. Samples (5 μl) of each culture were replica‐spotted onto LB agar, LB agar 2% SDS and TMAO plates each containing the appropriate antibiotics and incubated as previously described (Buchanan *et al*., 2002; Palmer *et al*., 2010). For measurement of TMAO reductase activity, strains were grown anaerobically overnight at 37°C in LB containing 0.4% (w/v) TMAO and 0.5% (v/v) glycerol. Periplasmic fractions were prepared using ethylenediamminetetraacetate (EDTA)/lysozyme treatment and TMAO:benzyl viologen oxidoreductase activity in the periplasmic fraction was measured as described previously (Palmer *et al*., 2010).

Bacterial two‐hybrid analyses were performed as described by Karimova *et al*. (1998; 2001). For quantitative assessment, β‐galactosidase assays were performed according to the method of Miller on strains grown to exponential phase at 30°C and permeabilised with toluene (Miller, 1992). Data shown are an average of two technical replicates performed on three biological replicates.

### BN‐PAGE

An overnight culture of the strains of interest was inoculated at 1:100 dilution into 50 ml of LB supplemented with appropriate antibiotics and incubated with shaking at 37°C until an OD_600nm_ of 0.6 was reached. All subsequent steps were undertaken at 4°C. Cells were pelleted by centrifugation, then washed with 50 mM Tris–HCl, pH 7.5 buffer and resuspended in 2 ml of the same buffer supplemented with EDTA‐free protease inhibitor (Roche). Cell suspensions were lysed by sonication and the lysates were clarified by centrifugation at 17,000 × *g* for 10 min. Membranes were pelleted by ultracentrifugation at 220,000 × *g* for 30 min and resuspended in 100 μl of 50 mM Tris–HCl pH 7.5 10% glycerol buffer. Total membrane protein was quantified using the DC Protein Assay kit (Bio‐Rad).

Membrane samples (400 mg of protein) were pelleted and then solubilised in 40 μl of Buffer A (50 mM NaCl, 50 mM imidazole, 2 mM 6‐aminohexanoic acid, 1 mM EDTA, pH 7.0) containing 2% digitonin for 30 min on ice. Unsolubilised material was pelleted by centrifugation at 220,000 × *g* for 20 min and the supernatant was removed and supplemented with 5% glycerol and 0.2% Coomassie Brilliant Blue. Blue native polyacrylamide gel electrophoresis (BN‐PAGE) on solubilised samples was carried out using precast gels (Novex Bis‐Tris NativePAGE mini gel 4–16%, Life Technologies) and buffers (anode, cathode B and cathode B10) as described in Wittig *et al*. (2006). Separated proteins were transferred to polyvinylidene difluoride membrane using an iBlot 1 dry transfer device (Life Technologies) and the membranes were destained with 25% methanol 10% acetic acid solution and pure methanol as described (Wittig *et al*., 2006). Immunoreactive bands were visualised using an anti‐TatC peptide antibody (raised in rabbits to the amino‐acid sequence GKGRNREEENDAEAESEKTEE) obtained from GenScript (Piscataway, USA) that was used at 1/5000 dilution, and an anti‐TatB antibody [also raised in rabbits (Sargent *et al*., 2001)] or a goat anti‐rabbit HRP conjugated antibody (BioRad) used at 1/10,000 dilution diluted at 1/1000, with the Clarity Western ECL Substrate Kit (BioRad) and recorded with the GeneGNOME camera (Syngene).

A small volume of the same solubilised membrane fractions were also analysed by denaturing SDS–PAGE (12% acrylamide) (Laemmli, 1970), by mixing with 1 × Lammli buffer. Proteins were transferred to nitrocellulose membranes using the iBlot dry transfer device and incubated with the same anti‐TatC (1/10,000 dilution) and anti‐TatB (1/1000 dilution) antibodies. Immunoreactive bands were visualised as before.

### Purification of TatC‐his and SufI‐his complexes

The purification of TatC‐his and SufI‐his‐containing complexes produced in strain DADE/pRep4 from the pUNITAT2 and pFAT75ΔASufIhis plasmids, respectively, or of chromosomally encoded TatC‐his from strain MC75CH was performed as described in Fritsch *et al*. (2012) with the following modifications: Buffer B (20 mM MOPS pH 7.2, 200 mM NaCl) was used as resuspension buffer; membrane protein was solubilised using 1% digitonin in Buffer B supplemented with 50 mM imidazole (25 mM imidazole for chromosomally encoded TatC‐his); Buffer B containing 50 mM imidazole (25 mM imidazole for chromosomally encoded TatC‐his) and 0.1% digitonin was used as wash buffer; and Buffer B containing 10 mM EDTA and 0.1% digitonin was used as elution buffer (as protein samples in high concentrations of imidazole run poorly for BN‐PAGE).

Samples from each step of the purification were diluted in 1× Lammli buffer, separated by SDS–PAGE (12% acrylamide) transferred to nitrocellulose membrane and immunoblotted with anti‐his‐HRP (Abcam, 1/50,000 dilution to detect TatC‐his and 1/20,000 to detect SufI‐his), anti‐TatC (1/10,000, to detect untagged TatC bound to SufI‐his) anti‐TatB (1/1000) and anti‐TatA [raised in rabbits and used at 1/10,000 dilution (Sargent *et al*., 2001)].

Goat anti‐rabbit and anti‐mouse HRP conjugated antibodies (BioRad) were used at 1/10,000 dilution.

### Disulphide crosslinking

*In vivo* disulphide cross‐linking experiments were performed using the *tat* ^−^ strain DADE, with Tat proteins produced from plasmid pTatBC101. For experiments also involving co‐overproduction of substrate protein, the strain additionally contained plasmid pQE80‐CueO (Leake *et al*., 2008). Fresh LB medium was inoculated with a 1:100 dilution of an overnight culture of the strains of interest, supplemented with 1 mM of isopropyl‐*β*‐D‐galactopyranoside to induce the expression of CueO‐his as required, and cultured aerobically at 37°C until an OD_600nm_ of 0.3 was reached. At this point, three 25 ml aliquots of each culture were withdrawn and each was supplemented with fresh LB medium to a final volume of 50 ml. One sample was left untreated as a control, a second was supplemented with copper–phenanthroline to a final concentration of 0.3 mM (oxidising conditions) and the third incubated with 10 mM dithiothreitol (reducing conditions). Cells were incubated at 37°C with agitation for 15 min, harvested, resuspended and free sulphydryls quenched by addition 1 ml of 20 mM Tris–HCl, pH 7.5, 200 mM NaCl, 12 mM EDTA, 8 mM *N*‐ethylmaleimide. Following supplementation with protease inhibitor cocktail (Roche), cell membranes were extracted using the protocol described earlier and resuspended in 40 μl of Buffer C (50 mM Tris–HCl, pH 7.5, 5 mM MgCl_2_, 10% glycerol). A sample of the supernatant following ultracentrifugation was retained as the soluble cell fraction. Fifty micrograms of total membrane protein and 20 μl of ultracentrifugation supernatant, both in 1× Lammli buffer, were separated by SDS–PAGE and blotted with anti‐TatC (1/10,000) and anti‐his non‐conjugated (1/2000) antibody (to detect his‐tagged CueO) respectively. A goat anti‐mouse HRP conjugated antibody (BioRad) was used at 1/10,000 dilution.

### Fluorescence microscopy

Cells were prepared for microscopy as previously described (Alcock *et al*., 2013). Cells were imaged by pseudo total internal reflection fluorescence imaging as previously described (Alcock *et al*., 2013), with the following modifications: A 532 nm, 50mW, solid‐state laser (Suwtech) was used, and excitation light focused at the back of an oil immersion objective lens (100×∼ Plan Apo N.A. 1.49; Nikon). The power entering the back of the objective was 10.8 mW, resulting in a power density in the image plane of 137 μW μm^−2^. A 532 nm LP emission filter was used (Semrock), and data were collected at 20 ms exposure times. For figure composition, image stacks were imported into MATLAB (MathWorks). Image stacks averaged over 40 ms were scaled to display 1500 arbitrary units (a.u.) as the minimum (black) and 7500 a.u. as the maximum (white) and were exported as PNG files. The CueO antibody used to analyse CueO levels in these experiments has been described previously (Alcock *et al*., 2013), and the Sigma anti‐FLAG M2 antibody was used to detect TatC‐FLAG.

### Homology modelling of *E* *. coli* TatC

Modeller 9v9 (Sali and Blundell, 1993) was used to create the homology model of *E. coli* TatC, based on the X‐ray structure of the *A. aeolicus* protein (PDB: 4B4A).

## Supporting information


Supporting Information
