# Supplementary material for: The TatC component of the twin‐arginine protein translocase functions as an obligate oligomer
Source: Mol Microbiol. 2015 Jul 22;98(1):111–29. doi: 10.1111/mmi.13106 (PMC5102672; doi:10.1111/mmi.13106)
Supplement: Supplementary file 1 — Supporting Information [file MMI-98-111-s001.pdf]

Plasmid	Description	Reference
pTAT1d	pUNI-PROM carrying *E. coli tatABC* with engineered restriction sites allowing facile replacement of each gene	Maldonado *et al*. (2011b)
pTTC1	pSU40 encoding TorA$_{ss}$-CAT fusion	Maldonado *et al*. (2011b)
pUNITAT2	*tatABC* operon in pQE60, *tatC* fused to 3' hexahistidine coding sequence.	McDevitt *et al*. (2005)
pTAT101	Very low copy number vector expressing *tatABC* from *tat* promoter. Kan^R	Kneuper *et al*. (2012)
pTATBC101	Very low copy number vector expressing *tatBC* from *tat* promoter. Kan^R	Alcock *et al.* (2013)
P101C*TatBC	Very low copy number vector expressing *tatBC* from *tat* promoter with a modified RBS. Kan^R	Alcock *et al.* (2013)
pFAT75ΔA	*tatBC* in pQE60, *tatC* fused to 3' hexahistidine coding sequence.	Tarry *et al*. (2009)
pREP4	Kan^R, *lacI*	Zamenhof and Villarejo (1972)
pQE80	Overexpression vector, Amp^R	Qiagen
pQE80-CueO	*cueO* cloned into overexpression vector pQE80.	Leake *et al*. (2008)
pTat1d D5E	As pTAT1d, TatC D5E exchange	This work
pTat1d T11A	As pTAT1d, TatC T11A exchange	This work
pTat1d F37S	As pTAT1d, TatC F37S exchange	This work
pTat1d P48L	As pTAT1d, TatC P48L exchange	This work
pTat1d P54L	As pTAT1d, TatC P54L exchange	This work
pTat1d M59K	As pTAT1d, TatC M59K exchange	This work
pTat1d V64E	As pTAT1d, TatC V64E exchange	This work
pTat1d S66P	As pTAT1d, TatC S66P exchange	This work
pTat1d K73E	As pTAT1d, TatC K73E exchange	This work
pTat1d L74P	As pTAT1d, TatC L74P exchange	This work
pTat1d F94R	As pTAT1d, TatC F94R exchange	This work
pTat1d V145E	As pTAT1d, TatC V145E exchange	This work
pTat1d S148P	As pTAT1d, TatC S148P exchange	This work
pTat1d D150G	As pTAT1d, TatC D150G exchange	This work
pTat1d D150Y	As pTAT1d, TatC D150Y exchange	This work
pTat1d F165L	As pTAT1d, TatC F165L exchange	This work
pTat1d G204R	As pTAT1d, TatC G204R exchange	This work
pTat1d M205R	As pTAT1d, TatC M205R exchange	This work
pTat1d D211R	As pTAT1d, TatC D211R exchange	This work
pTat1d Q215R	As pTAT1d, TatC Q215R exchange	This work
pTat1d C224R	As pTAT1d, TatC C224R exchange	This work
pTat1d Y42stop	As pTAT1d, TatC Y42 stop codon exchange	This work
pTat1d S185stop	As pTAT1d, TatC S185 stop codon exchange	This work
pTat1d E187stop	As pTAT1d, TatC 187 stop codon exchange	This work
pTat1d L189stop	As pTAT1d, TatC L189 stop codon exchange	This work
pTat1d Y195stop	As pTAT1d, TatC Y195 stop codon exchange	This work
pTat1d L218stop	As pTAT1d, TatC L218 stop codon exchange	This work
pTat1d BC fusion 5	As pTat1d, encoding TatBC fusion protein (amino acid sequence $S_{166(TatB)}SSDNLSMS_{2(TatC)}$	This work

pTat1d BC fusion 358	As pTat1d, encoding TatBC fusion protein (amino acid sequence $S_{166(TatB)}$SSDKLSMS$_{2(TatC)}$	This work
pTat1d BC fusion 364	As pTat1d, encoding TatBC fusion protein (amino acid sequence $S_{166(TatB)}$SSETLSMS$_{2(TatC)}$	This work
pTat1d BC fusion 5 Y42stop	As pTat1d BC fusion 5, TatC Y42 stop codon exchange.	This work
pTat1d BC fusion 5 E187stop	As pTat1d BC fusion 5, TatC E187 stop codon exchange.	This work
pTat1d BC fusion 5 L218stop	As pTat1d BC fusion 5, TatC L218 stop codon exchange.	This work
pTat1d BC fusion 358 A160V	As pTat1d BC fusion 358, TatC A160V codon exchange.	This work
pTat101 cys less	As pTat101, producing cys-less variant of TatC	This work
pTat101 A65C	As pTat101 cys less, TatC A65C exchange	This work
pTat101 G144C	As pTat101 cys less, TatC G144C exchange	This work
pTat101 S148C	As pTat101 cys less, TatC S148C exchange	This work
pTat101 M205C	As pTat101 cys less, TatC M205 exchange	This work
pTat101 A65C P48L	As pTat101 A65C, TatC P48L exchange	This work
pTat101 A65C S66P	As pTat101 A65C, TatC S66P exchange	This work
pTat101 A65C V145E	As pTat101 A65C, TatC V145E exchange	This work
pTat101 A65C D150Y	As pTat101 A65C, TatC D150Y exchange	This work
pTat101 G144C P48L	As pTat101 G144C, TatC P48L exchange	This work
pTat101 G144C M59K	As pTat101 G144C, TatC M59K exchange	This work
pTat101 G144C S66P	As pTat101 G144C, TatC S6P exchange	This work
pTat101 G144C V145E	As pTat101 G144C, TatC V145E exchange	This work
pTat101 G144C D150Y	As pTat101 G144C, TatC D150Y exchange	This work
pTat101 G144C M205R	As pTat101 G144C, TatC M205R exchange	This work
pTat101 S148C P48L	As pTat101 S148C, TatC P48L exchange	This work
pTat101 S148C M59K	As pTat101 S148C, TatC M59K exchange	This work
pTat101 S148C S66P	As pTat101 S148C, TatC S6P exchange	This work
pTat101 S148C V145E	As pTat101 S148C, TatC V145E exchange	This work
pTat101 S148C D150Y	As pTat101 S148C, TatC D150Y exchange	This work
pTat101 S148C M205R	As pTat101 S148C, TatC M205R exchange	This work
pTat101 M205C P48L	As pTat101 M205C, TatC P48L exchange	This work
pTat101 M205C M59K	As pTat101 M205C, TatC M59K exchange	This work
pTat101 M205C S66P	As pTat101 M205C, TatC S6P exchange	This work
pTat101 M205C V145E	As pTat101 M205C, TatC V145E exchange	This work
pTat101 M205C D150Y	As pTat101 M205C, TatC D150Y exchange	This work
pTatBC101 M205C	As pTatBC101, TatC M205C exchange	This work
pTatBC101 M205C P48L	As pTatBC101 M205C, TatC P48L exchange	This work
pTatBC101 M205C M59K	As pTatBC101 M205C, TatC M59K exchange	This work
pTatBC101 M205C S66P	As pTatBC101 M205C, TatC S66P exchange	This work
pTatBC101 M205C V145E	As pTatBC101 M205C, TatC V145E exchange	This work
pTatBC101 M205C D150Y	As pTatBC101 M205C, TatC D150Y exchange	This work

pUT18	Vector encoding T18 fragment of *B. pertussis cyaA* (amp^r)	Karimova *et al.* (2001)
pT25	Vector encoding T25 fragment of *B. pertussis cyaA* Cm^R	Karimova *et al.* (1998)
pUT18-TatC	pUT18 carrying *E. coli tatC*. Produces T18 with TatC fused C-terminally.	This work
pT25-TatC	pT25 carrying *E. coli tatC*. Produces T25 with TatC fused N-terminally.	This work
pUT18-TatC-P48L	As pUT18-TatC, P48L exchange	This work
pUT18-TatC-M59K	As pUT18-TatC, M59K exchange	This work
pUT18-TatC-S66P	As pUT18-TatC, S66P exchange	This work
pUT18-TatC-V145E	As pUT18-TatC, V145E exchange	This work
pUT18-TatC-D150Y	As pUT18-TatC, D150Y exchange	This work
pUT18-TatC-M205R	As pUT18-TatC, M205R exchange	This work
pT25-TatC-P48L	As pT25-TatC, P48L exchange	This work
pT25-TatC-M59K	As pT25-TatC, M59K exchange	This work
pT25-TatC-S66P	As pT25-TatC, S66P exchange	This work
pT25-TatC-V145E	As pT25-TatC, V145E exchange	This work
pT25-TatC-D150Y	As pT25-TatC, D150Y exchange	This work
pT25-TatC-M205R	As pT25-TatC, M205R exchange	This work
pUT18-TatC-M59K-M205R	As pUT18-TatC-M205R, M59K exchange	This work
pUT18-TatC-S66P-M205R	As pUT18-TatC-M205R, S66P exchange	This work
pUT18-TatC-V145E-M205R	As pUT18-TatC-M205R, V145E exchange	This work
pUT18-TatC-D150Y-M205R	As pUT18-TatC-M205R, D150Y exchange	This work
pT25-TatC-M59K-M205R	As pT25-TatC-M205R, M59K exchange	This work
pT25-TatC-S66P-M205R	As pT25-TatC-M205R, S66P exchange	This work
pT25-TatC-V145E-M205R	As pT25-TatC-M205R, V145E exchange	This work
pT25-TatC-D150Y-M205R	As pT25-TatC-M205R, D150Y exchange	This work
pUNITAT2 TatC P48L	As pUNITAT2, TatC P48L exchange	This work
pUNITAT2 TatC M59K	As pUNITAT2, TatC M59K exchange	This work
pUNITAT2 TatC S66P	As pUNITAT2, TatC S66P exchange	This work
pUNITAT2 TatC V145E	As pUNITAT2, TatC V145E exchange	This work
pUNITAT2 TatC D150Y	As pUNITAT2, TatC D150Y exchange	This work
P101C*TatBCflag	As P101C*TatBC, TatC produced with C-terminal FLAG epitope	This work
P101C*TatBCflag S66P	As P101C*TatBC with TatC S66P exchange	This work
P101C*TatBCflag D150Y	As P101C*TatBC with TatC D150Y exchange	This work
pFAT75ΔASufIhis	*tatBCsufI* in pQE60, *sufI* fused to 3' hexahistidine coding sequence.	This work
pFAT75ΔASufIhisKK	As pFAT75ΔASufIhis, but with sufI R5K, R6K variant	This work
pFAT75ΔAP48LSufIhis	As pFAT75ΔASufIhis, P48L exchange	This work
pFAT75ΔAS66PSufIhis	As pFAT75ΔASufIhis, S66P exchange	This work
pFAT75ΔAV145ESufIhis	As pFAT75ΔASufIhis, V145E exchange	This work

pFAT75ΔAD150YSufIhis	As pFAT75ΔASufIhis, D150Y exchange	This work
pFAT75ΔADF94AE103A SufIhis	As pFAT75ΔASufIhis, F94A and E103A exchanges	This work

Table S1. Plasmids used and constructed in this study.

Primer	Sequence
TatC_D5E_fwd	GCATGTCTGTCGACGAAACTCAACCGCTTATC
TatC_D5E_rev	GATAAGCGGTTGAGTTTCGTCGACAGACATGC
TatC_T11A_fwd	CTCAACCGCTTATCGCGCATCTGATTGAGC
TatC_T11A_rev	GCTCAATCAGATGCGCGATAAGCGGTTGAG
TatC_F37S_fwd	CCTGTGTCTGGTCTATTCCGCCAATGACATCTATC
TatC_F37S_rev	GATAGATGTCATTGGCGGAATAGACCAGACACAGG
C-P48Lf	GGTATCCGCGCTGTTGATCAAGC
C-P48Lr	GCTTGATCAACAGCGCGGATACC
C-P54Lf	CAAGCAGTTGCTGCAAGGTTCAACG
C-P54Lr	CGTTGAACCTTGCAGCAACTGCTTG
C-M59Kf	GGTTCAACGAAGATCGCCACCG
C-M59Kr	CGGTGGCGATCTTCGTTGAACC
C-V64Ef	GCCACCGACGAGGCCTCGCCGTTC
C-V64Er	GAACGGCGAGGCCTCGTCGGTGGC
C-S66Pf	CGACGTGGCCCCGCCGTTCTTTACG
TatC_K73E_fwd	GTTCTTTACGCCGATCGAGCTGACCTTTATGGTG
TatC_K73E_rev	CACCATAAAGGTCAGCTCGATCGGCGTAAAGAAC
C-L74Pf	CGCCGATCAAGCCGACCTTTATGG
C-L74Pr	CCATAAAGGTCGGCTTGATCGGCG
C-F94Rf	GGTGTGGGCACGCATCGCCCCAG
C-F94Rr	CTGGGGCGATGCGTGCCCACACC
C-V145Ef	CGGAAGGGGAACAGGTATCCAC
C-V145Er	GTGGATACCTGTTCCCCTTCCG
C-S148Pf	GTGCAGGTACCGACCGACATC
C-S148Pr	GATGTCGGTCGGTACCTGCAC
C-D150Gf	CAGGTATCCACCGGCATCGCCAGC
C-D150Gr	GCTGGCGATGCCGGTGGATACCTG
TatC_D150Y_fwd	GGGGTGCAGGTATCCACCTACATCGCCAGCTATTTAAGC
TatC_D150Y_rev	GCTTAAATAGCTGGCGATGTAGGTGGATACCTGCACCCC
TatC_F165L_fwd	GCGCTGTTTATGGCGCTTGGTGTCTCCTTTG
TatC_F165L_rev	CAAAGGAGACACCAAGCGCCATAAACAGCGC
C-G204Rf	GCATTCGTTGTCCGCATGTTGCTG
C-G204Rr	CAGCAACATGCGGACAACGAATGC
C-M205Rf	GTTGTCGGGAGGTTGCTGACG
C-M205Rr	CGTCAGCAACCTCCCGACAAC
TatC_D211R_fwd	CTGACGCCGCCGCGCGTCTTCTCGCAAAC
TatC_D211R_rev	GTTTGCGAGAAGACGCGCGGCGGCGTCAG
TatC_Q215R_fwd	GTCTTCTCGCGAACGCTGTTG
TatC_Q215R_rev	CAACAGCGTTCGCGAGAAGAC
TatC_C224R_F	GTTGGCGATCCCGATGTACCGTCTGTTTGAAATCGGTGTC
TatC_C224R_R	GACACCGATTTCAAACAGACGGTACATCGGGATCGCCAAC
BCfusion_5_F	CCCCTTCCTCGAGTGATAACTTAAGCATGTCTGTCG
BCFusion_5_R	CGACAGACATGCTTAAGTTATCACTCGAGGAAGGGG

BCfusion_358_F	CTTCCTCGAGTGATAAACTAAGCATGTCTGTCGACG
BCfusion_358_R	CGTCGACAGACATGCTTAGTTTATCACTCGAGGAAG
BCfusion_364_F	CCTTCCCCTTCCTCGAGTGAAACCTTAAGCATGTCTGTC
BCfusion_364_R	GACAGACATGCTTAAGGTTTCACTCGAGGAAGGGGAAGG
TatC_Y42stop_fwd	CGCCAATGACATCTAACACCTGGTATCCG
TatC_Y42stop_rev	CGGATACCAGGTGTTAGATGTCATTGGCG
TatC_S185stop_F	GCTGTGCTGGATGGGGATTACCTAGCCAGAAGACTTACGCAAAAAAC
TatC_S185stop_R	GTTTTTTGCGTAAGTCTTCTGGCTAGGTAATCCCCATCCAGCACAGC
TatC_E187stop_F	GGGGATTACCTCGCCATAAGACTTACGCAAAAAACGCC
TatC_E187stop_R	GGCGTTTTTTGCGTAAGTCTTATGGCGAGGTAATCCCC
TatC_Y195stop_F	CTTACGCAAAAAACGCCCGTAGGTGCTGGTTGGTGCATTCG
TatC_Y195stop_R	CGAATGCACCAACCAGCACCTACGGGCGTTTTTTGCGTAAG
TatC_L218stop_fwd	CTTCTCGCAAACGCTGTAGGCGATCCCGATGTACTG
TatC_L218stop_rev	CAGTACATCGGGATCGCCTACAGCGTTTGCGAGAAG
TatC6741	CGCATCTGATTGAGCTGCGTAAG
TatCstopApa	GCGCAGATCTGGGCCCTTATTCTTCAGTTTTTTCG
SufIstartApa	GCGCGGGCCCATTAAAGAGGAGAAATTAACCATGTCACTCAGTCGGCGTCAGTTC
SufIBglII	GCGCAGATCTCGGTACCGGATTGACCAAC
C-F94A1	GGTGTGGGCAGCGATCGCCCCAG
C-F94A2	CTGGGGCGATCGCTGCCCACACC
C-E103A-1	CGCTGTATAAGCATGCGCGTCGCCTGGTGGTGC
C-E103A-2	GCACCACCAGGCGACGCGCATGCTTATACAGCG
pUT18TatCforXba	GCGCTCTAGAGTCTGTAGAAGATACTCAACCGCTTATCAGC
pUT18TatCrevBam	GCGCGGATCCGCTTCTTCAGTTTTTTCGCTTTCTGCTTCAGC
pT25TatCforBam	GCGCGGATCCGTCTGTAGAAGATACTCAACCGCTTATCACGC
pT25TatCrevKpn	GCGCGGTACCGTTATTCTTCAGTTTTTTCGCTTTCTGCTTCAGC
p101C*BCflag_F	GAAAGCGAAAAAACTGAAGAAGACTACAAGGACGATGACAAGTAAGGCTGCAG GCATGCAAG
p101C*BCflag_R	CTTGCATGCCTGCAGCCTTACTTGTCATCGTCCTTGTAGTCTTCTTCAGTTTTT TCGCTTTC

Table S2. Oligonucleotides used in this study.
